# Extracellular vesicle-mediated transfer of CLIC1 protein is a novel mechanism for the regulation of glioblastoma growth

**DOI:** 10.18632/oncotarget.5105

**Published:** 2015-09-30

**Authors:** Matteo Setti, Daniela Osti, Cristina Richichi, Barbara Ortensi, Massimiliano Del Bene, Lorenzo Fornasari, Galina Beznoussenko, Alexandre Mironov, Germana Rappa, Alessandro Cuomo, Mario Faretta, Tiziana Bonaldi, Aurelio Lorico, Giuliana Pelicci

**Affiliations:** ^1^ Department of Experimental Oncology, European Institute of Oncology, Milan, Italy; ^2^ Department of Neurosurgery, Fondazione IRCCS Istituto Neurologico C. Besta, Milan, Italy; ^3^ Institute of Molecular Oncology (IFOM) of the Italian Foundation for Cancer Research (FIRC), Milan, Italy; ^4^ Cancer Research Center, Roseman University of Health Sciences with Roseman University College of Medicine, Las Vegas, NV, USA; ^5^ Department of Translational Medicine, Università del Piemonte Orientale, Novara, Italy

**Keywords:** glioblastoma, cancer stem cells, extracellular vesicles, cell proliferation, tumor growth

## Abstract

Little progresses have been made in the treatment of glioblastoma (GBM), the most aggressive and lethal among brain tumors. Recently we have demonstrated that Chloride Intracellular Channel-1 (CLIC1) is overexpressed in GBM compared to normal tissues, with highest expression in patients with poor prognosis. Moreover, CLIC1-silencing in cancer stem cells (CSCs) isolated from human GBM patients negatively influences proliferative capacity and self-renewal properties *in vitro* and impairs the *in vivo* tumorigenic potential. Here we show that CLIC1 exists also as a circulating protein, secreted via extracellular vesicles (EVs) released by either cell lines or GBM-derived CSCs. Extracellular vesicles (EVs), comprising exosomes and microvesicles based on their composition and biophysical properties, have been shown to sustain tumor growth in a variety of model systems, including GBM. Interestingly, treatment of GBM cells with CLIC1-containing EVs stimulates cell growth both *in vitro* and *in vivo* in a CLIC1-dose dependent manner. EVs derived from CLIC1-overexpressing GBM cells are strong inducers of proliferation *in vitro* and tumor engraftment *in vivo*. These stimulations are significantly attenuated by treatment of GBM cells with EVs derived from CLIC1-silenced cells. However, CLIC1 modulation appears to have no direct role in EV structure, biogenesis and secretion. These findings reveal that, apart from the function of CLIC1 cellular reservoir, CLIC1 contained in EVs is a novel regulator of GBM growth.

## INTRODUCTION

Glioblastoma (GBM) is the most aggressive among tumors of glial origin and represents, with a median patient survival of 14 months, a social and economic burden [1, 2]. The complexity in the study of GBM resides in its extremely heterogeneous nature at both the cellular and molecular levels. GBM is composed by a subpopulation of cancer stem cells (CSCs) able to fuel and sustain tumor development and progression, as well as tumor cells and non-neoplastic parenchymal cells, comprising vascular cells, microglia, peripheral immune cells, deeply intermingled throughout the tumor mass [3–5]. All these cells communicate with each other via the secretion and uptake of a number of factors that play a pivotal role in controlling the course of pathology. Among these, secreted extracellular vesicles (EVs) are a class of small bilayered particles (50–150 nm in size) that have been extensively studied in a variety of model systems for their ability to transfer their molecular cargoes to target cells, both locally and at a distance, influencing the tumor phenotype [6–8].

The first member of Chloride Intracellular Channel family (CLIC), namely CLIC1, holds pathological implications in a variety of different tumoral contexts [9–12], being involved in cell cycle progression [13, 14], cell motility regulation [15–18], resistance to pharmaceutical compounds [19]. We have recently demonstrated that CLIC1 is functionally active as a chloride channel in GBM CSCs and that inhibition of CLIC1, either by gene silencing using RNA interference or by *in vitro* incubation with a blocking antibody, slows GBM CSC proliferation and self-renewal and reduces *in vivo* tumorigenicity [20]. The recent identification of CLIC1 outside the cellular environment, in biological fluids like plasma [21] and serum [22, 23], urine [24] and in the medium of different cell lines often in association with secreted EVs [25–31], fostered the hypothesis that a circulating CLIC1 protein is detectable in the context of brain tumors in association to EVs, and it might potentiate the function of CLIC1 intracellular reservoir as a novel regulator of GBM growth.

## RESULTS

### CLIC1 protein is secreted by GBM cells *in vitro*

**Figure 1:**
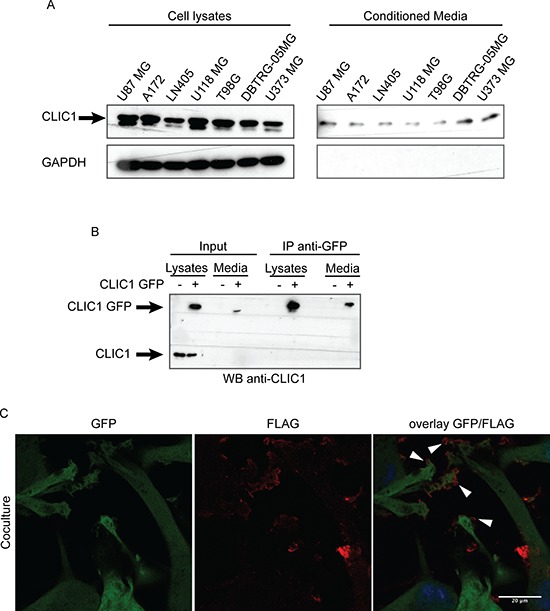
CLIC1 protein is secreted by glioblastoma cells **A.** CLIC1 expression was analyzed by Western blot in cell lysates (left panel) and conditioned media (right panel) of several GBM cell lines. GAPDH was used as loading control. A representative immunoblot is shown. **B.** U87 MG cells were mock infected or infected with CLIC1 GFP fusion protein. Cells lysates and conditioned media were immunoblotted (Input), or immunoprecipitated against GFP-tag (IP anti-GFP) and then immunoblotted with CLIC1 antibody. A representative immunoblot is shown. **C.** U87 MG cells expressing Green Fluorescent Protein (GFP) were co-cultured with U87 MG cells expressing the FLAG-tagged isoform of CLIC1 for 24 hours. Cells were stained using anti-FLAG antibody (red). Arrowheads mark FLAG-tagged isoform of CLIC1 on U87 MG GFP cells. Scale bar, 20 μm.

CLIC1 was recently identified by proteomic screens in the supernatants of various cell lines and in human fluids (i.e serum, plasma, urine) [21–24]. These intriguing observations prompted us to examine the possibility that CLIC1 protein could be released also by GBM cells. CLIC1 protein was expressed in the cell lysates of all GBM cell lines analyzed as well as in their conditioned media (Fig. 1A). The culture medium was devoid of GAPDH, suggesting that CLIC1 extracellular release was not a consequence of contamination by intracellular proteins (Fig. 1A). Moreover, cell viability measured by incorporation of propidium iodide (PI) was greater than 95%, indicating that CLIC1 release was not due to cell death (Fig. S1).

To further confirm CLIC1 protein release by GBM cells, we employed U87 MG cells overexpressing CLIC1 protein fused to Green Fluorescent Protein (GFP) at the N-terminal end (U87 CLIC1 GFP). 24 hours after cell plating, we collected culture medium and immunoprecipitated GFP-tagged CLIC1 protein. A single band of 50 kDa corresponding to CLIC1 GFP fusion protein was detected in the medium from U87 CLIC1 GFP cells, while no band was detected in the medium from mock transfected cells, confirming that exogenous CLIC1 GFP protein was released in the medium (Fig. 1B).

As a complementary approach, we co-cultured U87 MG cells expressing the FLAG-tagged isoform of CLIC1 (U87 CLIC1 FLAG) with U87 MG cells expressing Green Fluorescent Protein (U87 GFP). After 24 hours, we detected by immunofluorescence FLAG-tagged isoform of CLIC1 on U87 GFP cells (Fig. 1C), indicating that CLIC1 protein had been transferred from cell to cell. Collectively, these results demonstrate that CLIC1 protein is secreted by GBM cells *in vitro* and it is internalized by recipient cells.

### CLIC1 protein is secreted via extracellular vesicles (EVs)

**Figure 2:**
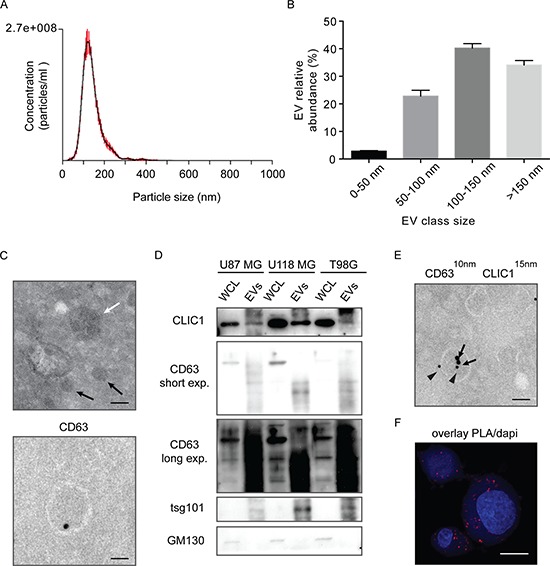
CLIC1 protein is secreted by GBM cells via Evs **A.** Size profile of EVs released by U87 MG cells measured by NTA. Shown the profile from a representative experiment. **B.** EVs were divided into four different dimensional classes, and their abundance was measured by NTA. Shown the profile from a representative experiment. **C.** Representative transmission electron micrograph of GBM cell-derived EVs (100 nm EVs, white arrow; 40 nm EVs, black arrows, Upper). Representative immuno-EM image of CD63 staining of GBM cell-derived EV (Lower). Scale bar, 100 nm. At least 200 EVs from 10 sections were examined in two independent experiments. **D.** Whole cell lysates (WCL) from different GBM cell lines and lysates from corresponding EVs were analysed for the indicated proteins by Western blotting. A representative immunoblot is shown. **E.** Representative immuno-EM image of GBM cell-derived EVs. Double-staining was conducted using anti-CD63 antibody (10 nm gold particles, indicated by arrowheads) and anti-CLIC1 antibody (15 nm gold particles, indicated by arrows) Scale bar, 110 nm. At least 300 EVs from 30 sections were examined in three independent experiments. **F.** CLIC1 and CD63 colocalization was assessed in U87 MG cells by *in situ* proximal ligation assay (PLA). Scale bar, 20 μm. At least 10 cells from 5 sections were examined in three independent experiments.

We then investigated the mechanism of CLIC1 protein release from GBM cells. Interestingly, CLIC1 protein encloses two structural features indicative of a vesicle-mediated secretion: a PP*X*Y motif for binding of Nedd4 type E3 ubiquitine ligases and a dileucine cluster motifs [32]. Notably, Nedd4-mediated ubiquitination is responsible for ubiquitinated protein sorting to specific endosome compartments prior to vesicle budding [33]. Of note, CLIC1 protein is ubiquitinated in GBM cells, as shown by immunoprecipitation with anti-CLIC1 antibody and immunoblot with antiubiquitin antibody (Fig. S2). In agreement, analysis of ExoCarta database revealed that CLIC1 protein resides in extracellular vesicles (EVs), comprising both exosomes and shedded microvesicles, released from different cell types [27]. To investigate whether CLIC1 secreted protein is contained in EVs from GBM cells, we selected U87 MG cells, which are known to produce significant amounts of EVs [8]. EVs were isolated from U87 MG conditioned media according to an established protocol based on serial centrifugation [34]. Nanoparticle Tracking Analysis (NTA) revealed the presence of an heterogeneous population of vesicles, with an average mean diameter of 146 nm (Fig. 2A), and comprising vesicles smaller than 50 nm (2,8% of the total), vesicles ranging in size from 50 to 100 nm (22,8% of the total) or from 100 to 150 nm (40,2% of the total), and vesicles larger than 150 nm (34,1% of the total) (Fig. 2B). Electron microscopy showed heterogeneous vesicles (Fig. 2C, upper panel) positive for CD63, a tetraspanin strongly enriched in late endosomes and exosomes (Fig. 2C, lower panel) [35]. Purified EVs were enriched in the exosome specific proteins CD63 and tsg101 (tumor susceptibility gene 101), as shown by immunoblotting analysis, while whole cell lysates were immuno-negative for both proteins (Fig. 2D). Notably, GM130 *cis*-Golgi marker was absent, demonstrating the purity of the isolated fractions (Fig. 2D). Interestingly, CLIC1 protein was expressed in EVs derived from U87 MG cells, as demonstrated by both immunoblot analysis (Fig. 2D) and immuno-electron microscopy (Fig. 2E). CLIC1 protein was also expressed in EVs derived from two other GBM cell lines (i.e. U118 MG and T98G) (Fig. 2D).

We next visualized the co-localization between CLIC1 and CD63 protein at the cellular level, by *in situ* proximal ligation assay (PLA) [36]. PLA revealed that a substantial fraction of CLIC1 protein was located in CD63-positive compartments of the cell, further supporting CLIC1 sorting to EVs (Fig. 2F and S3A). The possibility that PLA signals derived from non-specific binding of PLA probes was excluded by the absence of PLA fluorescence in CLIC1-silenced cells (Fig. S3B). Taken together, our data provide evidence that CLIC1 protein is secreted from GBM cells in EVs.

### CLIC1-containing EVs regulate the proliferative response of GBM cells

We previously described the role of CLIC1 protein in GBM progression through the modulation of GBM CSC self-renewal and proliferation [20]. Here, we sought to determine whether CLIC1-containing EVs are internalized by recipient cells and influence the proliferative response of GBM cells. To this end, we labeled U87 MG cell-derived EVs with the lipid-associating fluorescent dye PKH26. When PKH26-positive EVs were incubated with human embryonic kidney 293T cells, we observed a rapid uptake of labeled EVs into the recipient cells, as indicated by confocal microscopy (Fig. S4A) and flow cytometry (Fig. S4B). Labeled EVs displayed a time-dependent uptake kinetic, which reached the maximum 24 hours after incubation, when PKH26 fluorescence was observed in nearly 80% of recipient cells (Fig. S4B and S4C). Moreover, incubation at 4°C significantly reduced EV uptake (Fig. S4D).

**Figure 3:**
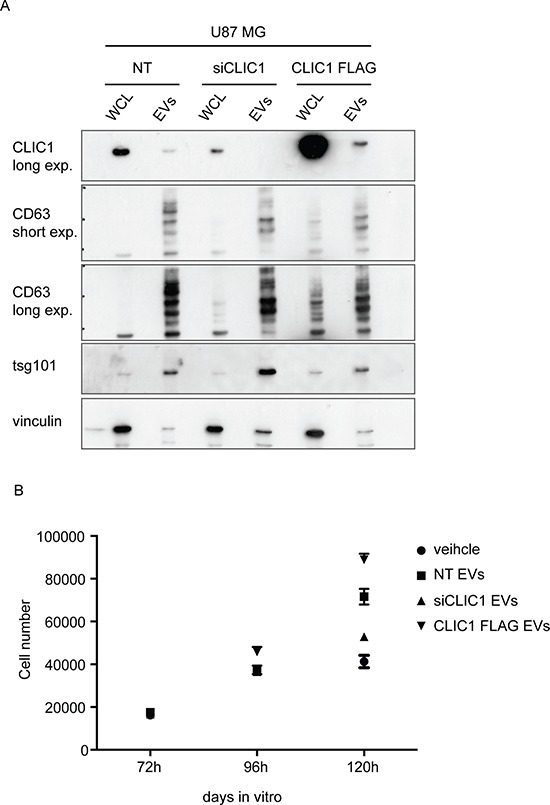
CLIC1-containing EVs regulate the proliferative response of GBM cells **A.** Whole cell lysates (WCL) obtained from U87 MG control cells (NT), CLIC1 silenced cells (siCLIC1) and CLIC1 overexpressing cells (CLIC1 FLAG), as well as lysates of corresponding EVs, were analysed by Western blotting using antibodies against CLIC1, and the EV markers CD63 and tsg101. Vinculin was used as loading control. A representative immunoblot is shown. **B.** U87 MG cells were incubated with EVs (50 μg/ml) derived from U87 MG NT, U87 MG siCLIC1 and U87 MG CLIC1 FLAG cells, or PBS as control (veichle). Cell growth was measured 72, 96 and 120 hours after EV administration. Three independent experiments were performed; error bars represent standard error;

To assess the effects of CLIC1-containing EVs on GBM cell growth, we silenced CLIC1 (U87 MG siCLIC1) or overexpressed a FLAG-tagged version of CLIC1 (U87 MG CLIC1 FLAG) (Fig. 3A) in U87 MG cells, which express endogenous CLIC1. We collected conditioned media from the same amount of control (U87 MG NT), U87 MG siCLIC1 and U87 MG CLIC1 FLAG cells and isolated EVs through serial centrifugations. Immunoblot analysis confirmed the modulated expression of CLIC1 in EVs derived from the three cell lines (Fig. 3A). Notably, CLIC1 expression level in EVs mirrored CLIC1 intracellular level (Fig. 3A). Moreover, the three EV groups expressed similar levels of the known exosomal markers CD63 and tsg101 (Fig. 3A). Functionally active EVs isolated from either U87 MG NT, U87 MG siCLIC1 and U87 MG CLIC1 FLAG cells were added to U87 MG recipient cells and cell proliferation was evaluated. Consistent with a previous report of growth stimulation induced by EVs on GBM cells [8], exposure of U87 MG cells to EVs isolated from U87 MG NT cells resulted in increased proliferation. Administration of an equal amount of EVs isolated from U87 MG CLIC1 FLAG cells showed a robust proliferative response of U87 MG recipient cells compared to untreated cells. Intriguingly, EV-mediated mitogenic stimulation was strongly impaired upon treatment with EVs derived from U87 MG siCLIC1 cells (Fig. 3B). Altogether, these data demonstrate that CLIC1-containing EVs modulate GBM proliferative response in a CLIC1-dependent fashion.

### CLIC1-containing EVs enhance GBM growth *in vivo*

**Figure 4:**
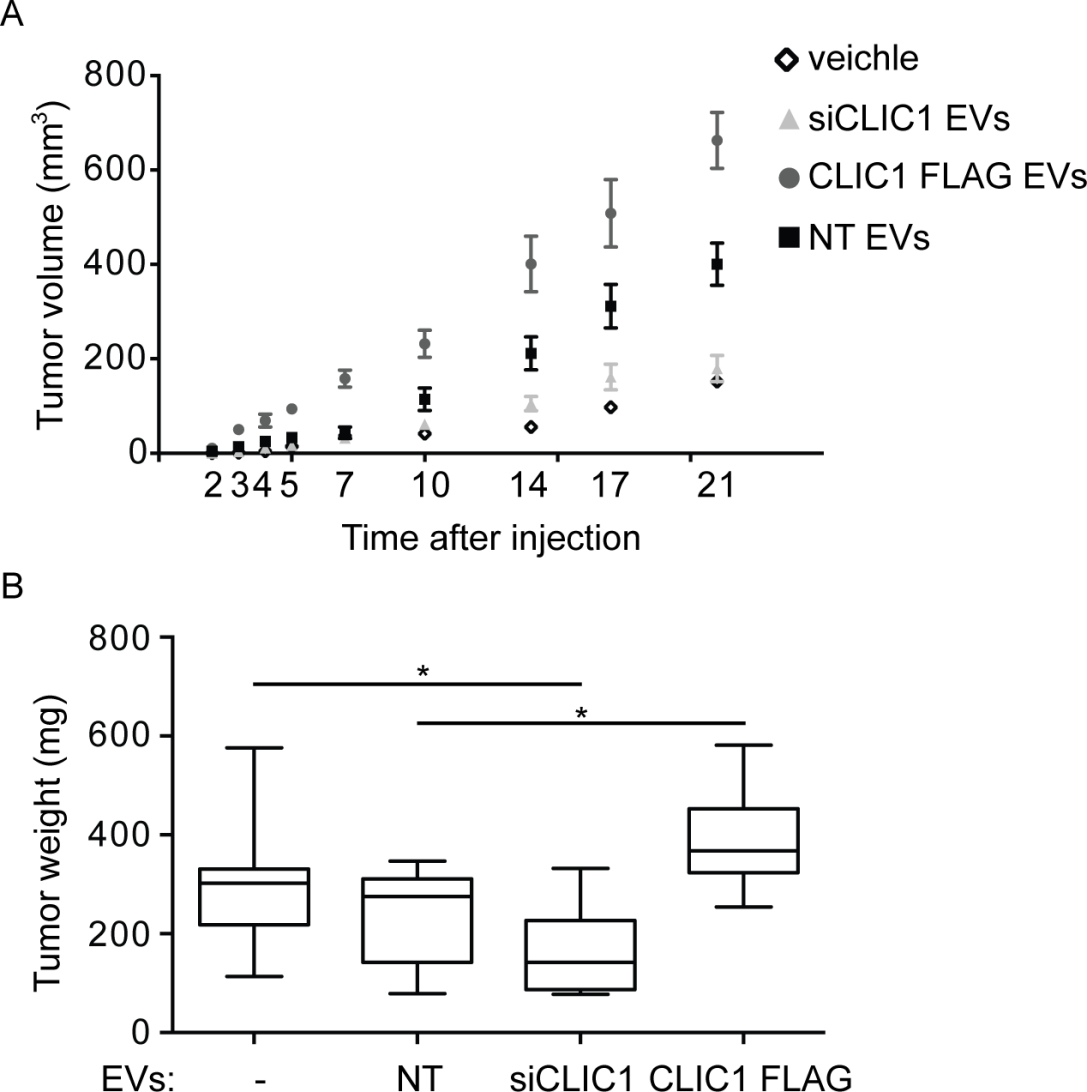
CLIC1-containing EVs enhance GBM growth *in vivo* U87 MG cells were incubated with EVs (1 μg/ml) derived from U87 MG NT, U87 MG siCLIC1 and U87 MG CLIC1 FLAG cells and subcutaneously injected into one flank of nude mice (*n* = 30 animals per group). As controls, untreated U87 MG cells were injected as well. Average tumor volumes at the indicated time points were measured. **B.** Tumors that formed after treatments described in A were surgically resected and weighted. Three independent experiments were performed. Error bars in A represent standard errors; solid lines in B are median values while error bars stand for minimum and maximum values. GLM tests of between-subjects effects showed statistically significant difference for the relative tumor growth according to the time, the treatment, and the interaction between those two variables. *p < 0.05.

To assess whether the effect of CLIC1-containging EVs occurred also *in vivo*, we injected U87 MG cells with NT EVs, siCLIC1 EVs and CLIC1 FLAG EVs, or PBS as control, subcutaneously into one flank of nude mice and monitored tumor growth over time. In agreement with the *in vitro* results, we observed a marked increase in tumor growth when we added NT EVs to the cells. Interestingly, co-injection of U87 MG cells with EVs isolated from U87 MG CLIC1 FLAG cell cultures resulted in a massive increase in tumor growth. On the contrary, the co-injection of U87 MG cells with EVs isolated from U87 MG siCLIC1 cells resulted in total abrogation of such enhancement (Fig. 4A). Approximately 3 weeks after cell implantation, we surgically resected the tumors and weighted them. As shown in Fig. 4B, the mean tumor weights were significantly higher in mice bearing tumors derived from U87 cells treated with CLIC1 FLAG EVs (Fig. 4B).

We next established orthotopic xenografts by injecting U87 MG cells together with NT EVs, siCLIC1 EVs, CLIC1 FLAG EVs, or PBS as control into brains of nude mice. Mice were sacrificed after one and three weeks and tumor incidence evaluated. The results indicated that EVs enriched in CLIC1 protein (NT EVs and CLIC1 FLAG EVs), but not those with low-CLIC1 content (siCLIC1 EVs), formed tumors with higher incidence (Table 1). These results collectively suggest that CLIC1 modulation influences EV-mediated tumorigenic potential of GBM cells.

**Table 1:** U87 MG cells were treated with NT EVs, siCLIC1 EVs and CLIC1 FLAG EVs and intracranially injected into nude mice (*n* = 10 animals per group)

Treatment	Engrafted tumors	
	1^st^ week	3^rd^ week
veichle	2/10	9/10
NT EVs	5/10	8/10
siCLIC1 EVs	1/10	2/10
CLIC1 FLAG EVs	8/10	10/10

As controls, untreated U87 MG cells were injected as well (*n* = 10). Tumor incidence was calculated.

### CLIC1 protein is contained in GBM Stem Cells-derived EVs

**Figure 5:**
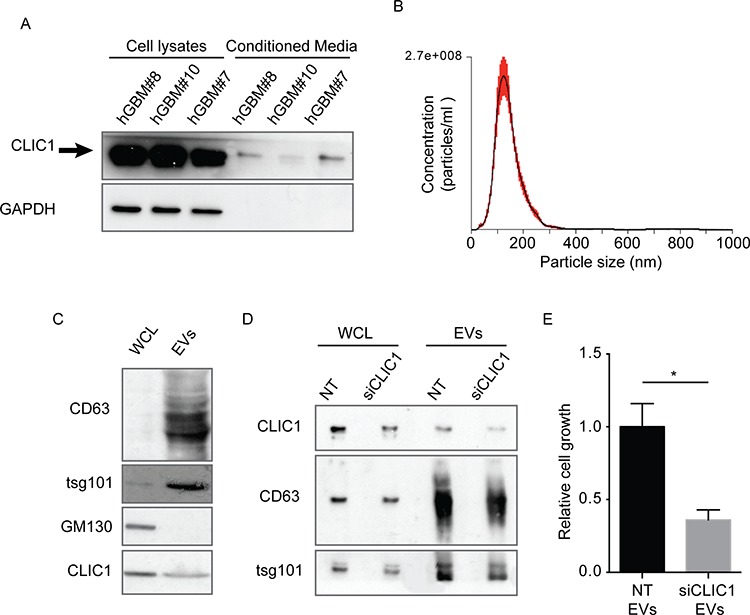
CLIC1 secreted protein resides in EVs released from GBM CSCs **A.** Representative immunoblots showing the expression of CLIC1 in three different GBM CSCs and in their respective media. GAPDH was used as loading control. **B.** Size profile of GBM CSC-derived EVs measured by NTA. Shown is the profile from a representative experiment. **C.** hGBM#10 CSC whole cell lysates (WCL) and lysates from corresponding EVs were analysed for the indicated proteins by Western blotting. A representative immunoblot is shown. **D.** Whole cell lysates (WCL) obtained from control hGBM#10 CSCs (NT) and CLIC1 silenced hGBM#10 CSCs (siCLIC1), as well as lysates of corresponding EVs, were analysed by Western blotting using antibodies against CLIC1, and the EV markers CD63 and tsg101. A representative immunoblot is shown. **E.** hGBM#10 CSCs were cultured in presence of EVs (50 μg/ml) derived from either NT or siCLIC1 hGBM#10 CSCs for 120 hours and assessed for cell growth by 3-(4, 5-dimethylthiazol-2-yl)-2, 5-diphenyltetrazolium bromide (MTT) assay. Three independent experiments were performed; error bars represent standard error; *p < 0.05.

GBM is maintained by a sub-population of CSCs which survive traditional therapies, allowing tumor regrowth, and explains the intratumoral cellular heterogeneity typical of this tumor [37]. We verified whether CLIC1 protein could be secreted from GBM CSCs, and whether its release occurred via EVs. GBM CSCs were isolated from human patient-derived GBMs and cultured in serum-free medium [38]. Examination of conditioned medium after 48 hour-cultures revealed that GBM CSCs secrete CLIC1 protein (Fig. 5A). Notably, CLIC1 was not released in the medium as a consequence of cell death, as demonstrated by the absence of GAPDH in the culture medium (Fig. 5A) and by the low percentage of cell death measured by the method of PI incorporation (Fig. S5). Next, we isolated EVs from the conditioned medium of GBM CSCs. The size of the isolated EVs was confirmed by NTA and ranged from 50 nm to 220 nm, with a mean value of 120 nm (Fig. 5B). Immunoblot analysis performed on EV extracts confirmed the enrichment of the exosomal markers CD63 and tsg101 and the lack of expression of GM130 *cis-*Golgi marker compared to the corresponding whole cell lysates (Fig. 5C). In agreement with data obtained from GBM cell lines, we confirmed that CLIC1 protein was expressed in GBM CSCs-derived EVs (Fig. 5C). The presence of CLIC1 in EVs was confirmed in CSCs established from a different GBM patient (Fig. S6). To investigate the role of CLIC1 in GBM CSC-derived EVs, we silenced CLIC1 expression in GBM CSCs (siCLIC1GBM CSCs) and purified EVs from culture medium by serial centrifugation. We observed a significant decrease of CLIC1 protein content in either siCLIC1GBM CSCs and in the corresponding EVs (siCLIC1 EVs) (Fig. 5D and S6). To determine the effect of CLIC1 reduction in EVs secreted by GBM CSCs, we incubated GBM CSCs with NT EVs and siCLIC1 EVs released by control GBM CSCs and siCLIC1 GBM CSCs, respectively. In agreement with the results obtained with GBM cells lines, CLIC1 depletion in EVs resulted in the reduction of GBM CSCs growth compared to control (Fig. 5E and S6). In the same way, intracranial injection of GBM CSCs treated with siCLIC1 EVs resulted in a significant reduction of tumor incidence at the early time point analyzed (i.e. 2 weeks after cell injection) (Table 2). Unexpectedly, the fact that all mice developed tumors at the end point of the experiment (i.e. 4 weeks after cell injection) reflects a delay in tumor formation in the early fase of GBM growth (Table 2).

**Table 2:** GBM CSCs were treated with their own NT EVs and siCLIC1 EVs, and intracranially injected into nude mice (*n* = 10 animals per group at the early time point analyzed, *n* = 5 animals per group at the end point of the experiment)

Treatment	Engrafted tumors	
	2^nd^ week	4^th^ week
NT EVs	6/10	4/5
siCLIC1 EVs	3/10	5/5

Tumor incidence was calculated.

### CLIC1 modulation in the cell does not alter EV phenotypic features and release

**Figure 6:**
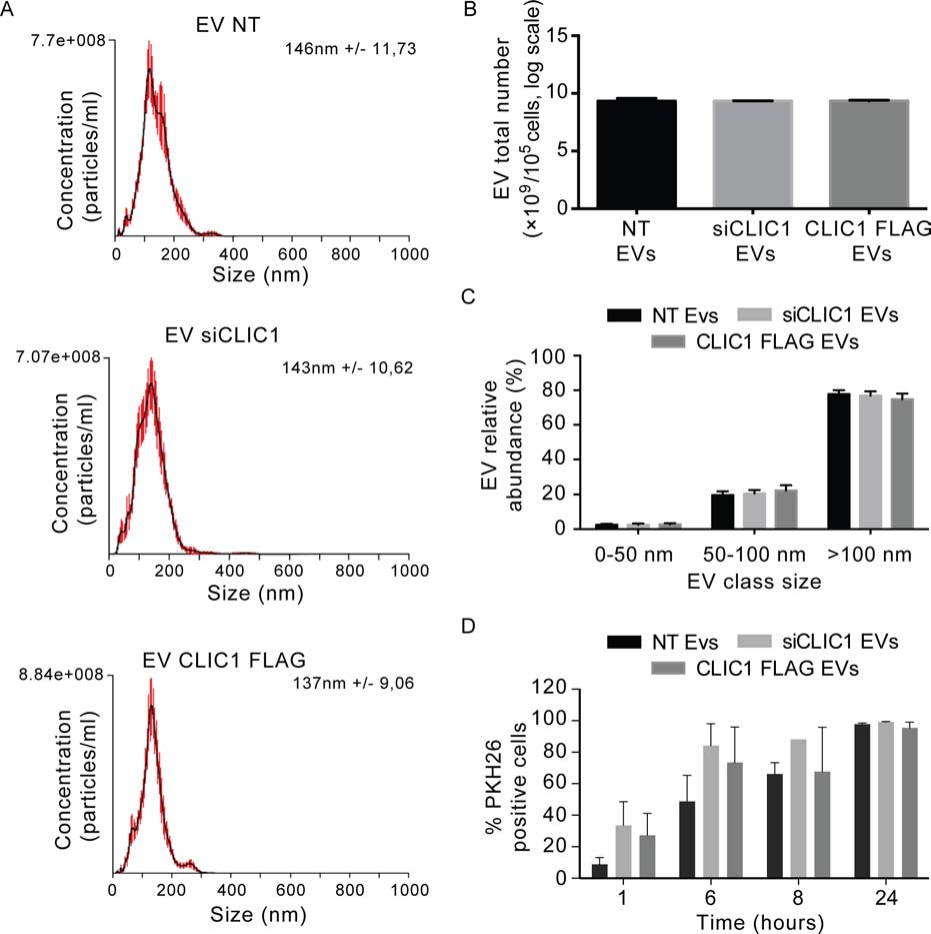
EV size and yield are not affected by CLIC1 modulation in GBM cells **A.** Size distribution of EVs shedded by U87 MG NT, U87 MG siCLIC1 and U87 MG CLIC1 FLAG cells, measured by NTA. **B.** The yield of EVs shedded by U87 MG NT, U87 MG siCLIC1 and U87 MG CLIC1 FLAG cells, measured by NTA. **C.** EVs were divided into three different dimensional classes, and their abundance was measured by NTA. A-C data are from a representative experiment. **D.** EVs derived from U87 MG NT, U87 MG siCLIC1 and U87 MG CLIC1 FLAG cells were labelled with PKH26 dye, and added to U87 MG recipient cells for 24 hours. EV uptake efficiency was expressed as percentage of PKH26 positive cells measured at different time points. Two-way ANOVA. Contribution of “treatment” and “time”: *p < 0.05. Results shown in B-D represent mean ± standard errors from three independent experiments.

We next sought to define whether the difference in EV stimulatory capacity might be due to changes in EV characteristics. EVs purified from the same number of U87 MG NT, U87 MG siCLIC1 and U87 MG CLIC1 FLAG cells were analyzed by NTA. Neither CLIC1 silencing nor CLIC1 overexpression in U87 MG cells had any effect on EV size distribution (Fig. 6A). Also, the yield of EVs secreted by U87 MG cells with modulated CLIC1 expression was identical to that of EVs produced by control cells (Fig. 6B). Notably, the abundance of either smaller vesicles (under 50 nm) or larger vesicles (ranging in size from 50 to 100 nm or larger then 100 nm) was not affected by CLIC1 modulation (Fig. 6C). Comparable results were obtained by analyzing EVs derived from T98G cells (Fig. S7) or from GBM CSC samples (Fig. S8). Moreover, CLIC1 modulation did not induce marked alterations in the expression of the canonical exosome markers CD63 or tsg101 either in EV samples (Fig. 3A, 5D and S6) or in GBM cells (Fig. S9). These data show that CLIC1 modulation in GBM cells does not affect EV phenotypic features, biogenesis or release of EVs. In addition, PKH26-labeled EVs from control, CLIC1-silenced and CLIC1-overexpressing U87 MG cells were equally taken up, thus excluding the possibility that the different proliferative response could be due to differences in EV uptake efficiency (Fig. 6D). Next, we used shotgun proteomics to determine the global protein composition of NT EVs, siCLIC1 EVs and CLIC1 FLAG EVs purified from U87 MG cells and thus assess whether differences in protein content could account for alteration in the proliferative response of GBM cells. We obtained a total of 2642, 2310 and 2328 proteins from NT EVs, siCLIC1 EVs and CLIC1 FLAG EVs, respectively (Fig. S10A and Supplementary Table 1). The proteomes of NT EVs, siCLIC1 EVs and CLIC1 FLAG EVs annotated upon high-resolution LCMSMS analysis are highly overlapping (Fig. S10B) and GO analysis did not reveal major alterations in EV protein content (Fig. S10C), thus reinforcing the idea that the pro-proliferative response induced by CLIC1 FLAG EVs can be mostly ascribed to CLIC1.

## DISCUSSION

Cancer development and progression depends on intercellular communication within cancer cells and between cancer cell and the surrounding stroma. Apart from direct cell-cell interactions and classical paracrine signals, extracellular vesicles (EVs) have emerged as a novel mediator of such intercellular communication by inducing multiple biological responses, i.e. tumor growth, tumor cell invasion and metastasis, angiogenesis, immune system response and drug resistance.

GBM cells adopt EV-mediated communication either to sustain their own proliferative and invasive potential or to modulate surrounding cell behaviour in order to promote tumor growth.

Here we provide evidence for the first time that Chloride Intracellular Channel 1 (CLIC1) protein, apart from its intracellular role, exists also as a secreted protein, released by GBM cells, either cell lines or patient-derived CSCs. Moreover, we demonstrate that its release occurs via EVs, and CLIC1 maintains its activity within EVs, i. e. modulating EV-mediated GBM cell proliferation in a CLIC1-dependent fashion.

Different lines of evidence support CLIC1 protein secretion by GBM cells. By biochemical approaches we detected CLIC1 protein in GBM cell culture media; moreover, by directly tracking exogenous CLIC1 protein (i.e. FLAG-tagged form of CLIC1 protein) *in vitro*, we demonstrated that CLIC1 protein can be secreted by a GBM donor cell and can efficiently enter into a different GBM recipient cell.

A significant part of secreted proteins exploits endoplasmic reticulum (ER)/Golgi pathway in order to be released in the extracellular environment. To do that, a target protein has to show a specific signal peptide that fates it to the exocytic pathway [39]. CLIC1 is not endowed with such region and it is synthetized by cytosolic ribosomes [40]. Thus, CLIC1 protein might exploit mechanisms of export alternative to canonical ER/Golgi secretion pathways [41]. This evidence is even strengthened by the lack of any colocalization between CLIC1 and ER/Golgi/lysosome markers [42].

The main trait of CLIC1 protein resides in its dual nature as a soluble globular cytoplasmic protein and as an integral membrane protein endowed with ion channel activity. The vast majority of CLIC1 protein is kept in the cytoplasm in its soluble form, as demonstrated by digitonin extraction: digitonin-resistant CLIC1 is either plasma membrane-bound or bound to cytosolic vesicles [42]. Moreover, CLIC1 vesicular localization is endorsed by CLIC1 expression pattern itself, which is dotted and scattered throughout the cell as reported in different human cell lines (PancI, HeLa, macrophages) [40, 42, 43]. Similarly, we observed that CLIC1 is expressed either at the plasma membrane or in punctate cytoplasmic structures in GBM cells ([20] and our unpublished data). The above observations prompted us to investigate the EV-mediated mechanism of CLIC1 protein release. GBM cell lines and primary human GBM samples release EVs *in vitro* [8] and *in vivo* [44]. Thus, we isolated EVs from cell culture media of either GBM cell lines and GBM CSCs by serial ultracentrifugation [34]. Sample purity was assessed by: (1) western immunoblot, showing a significant enrichment of the common endocytic markers CD63 and tsg101, (2) Nanoparticle Tracking Analysis proving that purified vesicle size fall in the 50–150 nm range, (3) EM analysis proving that EV samples comprise morphologically heterogeneous, bilayer-enclosed vesicles expressing CD63 protein. Interestingly, we found CLIC1 protein expressed in GBM cell-derived EVs in association with typical exosomal proteins, as demonstrated by western immunoblot, immune-EM and PLA technique. Supporting this finding, CLIC1 protein expression was documented within EVs released by a plethora of cell types and biological fluids [24, 25, 29–31]. In addition, CLIC1 is listed as a top-scored protein in *ExoCarta* database. Of further interest, CLIC1 protein was recently found circulating in human serum or plasma, and proposed as a potential biomarker in nasopharyngeal [21] and ovarian carcinoma [22]. CLIC1 primary structure harbors a PP*X*Y motif, recognized by the WW domain of the Nedd4 E3 ubiquitin-ligase [45, 46], and two dileucine motifs that are thought to support its enrollment into endocytic pathway [47, 48]. Considering that ubiquitination represents a major signal triggering the recruitment of ESCRT machinery and cargo incorporation within EVs [49, 50], the two structural hints existing in CLIC1 protein sequence, together with our biochemical data showing ubiquitinated CLIC1 in GBM cells, foster CLIC1 expression within EVs isolated by human GBM cells.

Recently, we have demonstrated that CLIC1 expression is associated with poorer prognostic GBMs, and its inhibition, by silencing its expression or blocking its channel activity, regulates GBM progression by targeting GBM CSCs properties [20].

EVs have been shown to sustain tumor growth in a variety of model systems, including GBM [8]. Interestingly, treatment of GBM cells with CLIC1-containing EVs stimulates cell growth both *in vitro* and *in vivo*. Either CLIC1 silencing or CLIC1 overexpression in GBM cells result in the secretion of EVs with reduced or increased CLIC1 protein content, respectively. Notably, EVs derived from CLIC1-overexpressing GBM cells accelerate cell growth *in vitro* and tumor engraftment *in vivo*. By contrast, these stimulations are significantly attenuated by GBM cell treatment with EVs that contained reduced CLIC1 protein content. However, modulation of CLIC1 protein expression in GBM cells does not significantly modify EV secretion or uptake, suggesting that the observed phenotype depends on EV molecular cargo modifications. Of note, proteomic approach reveals no major differences other than CLIC1 in EV protein cargo.

Our findings comprehensively suggest that CLIC1 is secreted within GBM cell-derived EVs, where it is involved in regulating EV-mediated pro-tumorigenic response. Of note, GBM is a heterogeneous tumor hierarchically organized, containing a small fraction of CSCs, responsible for tumor maintenance, progression and resistance to therapy, and giving rise to the bulk of malignant differentiated cells [51, 52]. Interestingly, both GBM cell lines and GBM CSCs are equally responsive to the treatment of EVs carrying distinct level of CLIC1 protein. Thus, it is conceivable that the CLIC1 pro-tumorigenic phenotype described previously [20] might rely also on CLIC1 circulating fraction other than CLIC1 cytoplasmic levels. Secreted CLIC1 protein may support tumor proliferation by (i) directly mediating the expansion of tumor cells, either bulk differentiated cells or CSCs, and (ii) molding tumor microenvironment. However, further studies are required in order to clarify how CLIC1 protein contained in EVs contributes to tumor microenvironment remodeling. [53]

## MATERIALS AND METHODS

Extensive description of cell culture conditions, EV isolation, western blotting analysis, nanoparticle tracking analysis, electron microscopy, as well as functional *in vitro* and *in vivo* assays, is provided in Supplementary Material.

### Human GBM samples

This study was approved by the Ethical Committee for human experimentation of IEO (European Institute of Oncology) and all patients signed an approved consent document prior to surgery. Surgical specimens of tumors were collected at the Neurosurgery Dpt. at IRCCS Istituto Clinico Humanitas and examined by a neuropathologist to verify that each case met criteria for GBM and to select a tissue fragment with high content of viable tumor tissue. Each tissue specimen was dissociated into single cell suspension and maintained as neurospheres in growth factors supplemented DMEM-F12 1:1 medium, as previously described [38]. Human GBM cell lines U87MG, A172, LN405, U118MG, T98G, DBTRG-05MG and U373 MG were purchased from American Type Culture Collection (ATCC) and maintained in DMEM (Lonza) supplemented with 2 mM glutamine, 100 U/mL penicillin and 100 μg/mL streptomycin, and 10% FBS. All cell cultures were maintained at 37°C in a humidified 5% CO_2_ incubator.

### Isolation of extracellular vesicles

GBM cells were grown in in serum-free medium for 48 hours prior to EV isolation. The media were then collected and processed according to standard procedures [34]. Briefly, media underwent serial centrifugation (500 g for 10′, 1200 g for 20′, 10000 g for 30′), they were filtered with a 0.22-μm pore filtersyringe, and then ultracentrifuged at 100000 g for 60′. The resulting pellet was washed in PBS before ultracentrifugation at 100000 g for 60′.

### Electron microscopy (EM)

For routine electron microscopy (EM), purified EVs were fixed with 1% glutaraldehyde for 1 hour, washed, post-fixed with 1% reduced osmium tetroxide for 1 hour, washed, post-stained with 0,3% thiocarbohydrazide; refixed in the OsO4 and embedded into Epon. Ultrathin sections were placed on formvar-coated grids or slot-grids. Immune-EM analysis was performed as previously described [54, 55].

### Nanoparticle tracking analysis (NTA)

We used the light-scattering characteristics of 488 nm laser light on EVs preparations undergoing Brownian motion injected by continuous flow into the sample chamber of an LM10 unit (Nanosight, Amesbury, UK). Three videos of 60–90 seconds were recorded of each sample. Data analysis was performed with NTA 3.0 software (Nanosight). The diffusion coefficient and hydrodynamic radius were determined using the Stokes–Einstein equation, and results were displayed as a particle size distribution. Data are presented as the average and standard deviation of the three video recordings. Since NTA is most accurate between particle concentrations in the range of 2 × 10^8^ to 2 × 10^9^/ml, when samples contained higher numbers of particles, they were diluted before analysis and the relative concentration calculated according to the dilution factor. Control 100 and 200 nm beads were supplied by Nanosight. NTA of a small sample of any given preparation revealed that they were essentially monodisperse, excluding the problem of aggregation, which may significantly impact on a biological system.

### Proximal ligation assay (PLA)

Samples were processed for PLA according to manufacturer's instructions (OLink Bioscience, Sweden) using the DuoLink *in situ* Orange detection reagent. Primary antibodies employed for PLA were: CD63 (mouse monoclonal, 1:50, clone FC-5.01 18–7300 Invitrogen), CLIC1 (rabbit polyclonal, 1:500, sc-134859 Santa Cruz, CA, USA).

### Western blot analysis

Primary antibodies: CLIC1 (mouse monoclonal, 1:1000, clone H-48, sc-134859, Santa Cruz, CA, USA), Vinculin (mouse monoclonal, 1:10000, clone HVIN-1, Sigma Aldrich, St. Louis, MO), CD63 (mouse monoclonal, 1:50, clone FC-5.01 18-7300 Invitrogen), tsg101 (goat polyclonal, 1:1000, sc-6037 Santa Cruz, CA, USA), GM130 (mouse monoclonal, 1:500, 610822, Becton Dickinson, Franklin Lakes, NJ, USA).

### *In vitro* cell growth

U87 MG were harvested in 96-well plates at the density of 5000 cells. Growth curves were determined by counting the cell numbers 72, 96 and 120 hours after EV administration.

GBM CSCs were harvested at a density of 3000 cells per well in 50 μl and treated with 50 μg/ml EVs. Cell viability was assessed after 120 hours of incubation. 3-(4, 5-dimethylthiazol-2-yl)-2, 5-diphenyltetrazolium bromide (MTT, 50 mg/ml) was added and, after incubation for 4 hours, crystals were dissolved in DMSO. Cell viability was evaluated by CellTiter 96^®^ AQueous Non-Radioactive Cell Proliferation Assay (Promega, Madison, WI). Three independent replicates were considered for each experiment.

### Animal experiments

Experiments involving animals were performed in accordance with the Italian Laws (D.L.vo 116/92 and following additions), which enforces EU 86/609 Directive (Council Directive 86/609/EEC of 24 November 1986 on the approximation of laws, regulations and administrative provisions of the Member States regarding the protection of animals used for experimental and other scientific purposes).

CD-1 nude mice (5 weeks old, Charles River Laboratories) were inoculated via s.c. injection on the dorsal region with U87 MG cells (2.5 ∼ 10^6^ in 150 μl of PBS) with or without EVs (1 μg/ml). Tumor mass was determined by caliper, measured in two perpendicular diameters and calculated using the formula 1/2*a* × *b*^2^, where *a* stands for the long diameter and *b* is the short diameter. Mice were sacrificed and tumors were collected for further analysis. For the intracranial orthotopic model (38), GBM cells (either U87 MG cells or GBM CSCs) were resuspended in 2 μl of PBS with or without EVs (1 μg/ml), and stereotaxically injected into the nucleus caudatus (coordinates: 0.7 - 1 mm posterior, 3 mm left lateral, 3.5 mm in depth from the dura) of 5 weeks old CD-1 nude mice. The mice were maintained until development of neurologic signs and then killed for tumor areas analysis using ImageJ software.

### Statistical analysis

Statistical analysis was performed using Statistical Package for Social Science (SPSS) software. Statistical significance of differences for all parametric variables has been tested by means of analysis of variance (ANOVA) with Bonferroni's correction. Data are graphed as mean ± standard error from at least three independent experiments. Differences were considered statistically significant when P was less than .05.

## SUPPLEMENTARY FIGURES AND TABLE





## References

[1] Dolecek TA, Propp JM, Stroup NE, Kruchko C (2012). CBTRUS statistical report: primary brain and central nervous system tumors diagnosed in the United States in 2005–2009. Neuro-oncology.

[2] Stupp R, Mason WP, van den Bent MJ, Weller M, Fisher B, Taphoorn MJ, Belanger K, Brandes AA, Marosi C, Bogdahn U (2005). Radiotherapy plus concomitant and adjuvant temozolomide for glioblastoma. The New England journal of medicine.

[3] Wu A, Wei J, Kong LY, Wang Y, Priebe W, Qiao W, Sawaya R, Heimberger A.B. (2010). Glioma cancer stem cells induce immunosuppressive macrophages/microglia. Neuro-oncology.

[4] Zhu TS, Costello MA, Talsma CE, Flack CG, Crowley JG, Hamm LL, He X, Hervey-Jumper SL, Heth JA, Muraszko KM (2011). Endothelial cells create a stem cell niche in glioblastoma by providing NOTCH ligands that nurture self-renewal of cancer stem-like cells. Cancer research.

[5] Calabrese C, Poppleton H, Kocak M, Hogg TL, Fuller C, Hamner B, Oh EY, Gaber MW, Finklestein D, Allen M (2007). A perivascular niche for brain tumor stem cells. Cancer cell.

[6] Pan BT, Teng K, Wu C, Adam M, Johnstone RM (1985). Electron microscopic evidence for externalization of the transferrin receptor in vesicular form in sheep reticulocytes. The Journal of cell biology.

[7] Booth AM, Fang Y, Fallon JK, Yang JM, Hildreth JE, Gould SJ (2006). Exosomes and HIV Gag bud from endosome-like domains of the T cell plasma membrane. The Journal of cell biology.

[8] Skog J, Wurdinger T, van Rijn S, Meijer DH, Gainche L, Sena-Esteves M, Curry WT, Carter BS, Krichevsky AM, Breakefield XO (2008). Glioblastoma microvesicles transport RNA and proteins that promote tumour growth and provide diagnostic biomarkers. Nature cell biology.

[9] Chen CD, Wang CS, Huang YH, Chien KY, Liang Y, Chen WJ, Lin KH (2007). Overexpression of CLIC1 in human gastric carcinoma and its clinicopathological significance. Proteomics.

[10] Huang JS, Chao CC, Su TL, Yeh SH, Chen DS, Chen CT, Chen PJ, Jou YS (2004). Diverse cellular transformation capability of overexpressed genes in human hepatocellular carcinoma. Biochemical and biophysical research communications.

[11] Petrova DT, Asif AR, Armstrong VW, Dimova I, Toshev S, Yaramov N, Oellerich M, Toncheva D (2008). Expression of chloride intracellular channel protein 1 (CLIC1) and tumor protein D52 (TPD52) as potential biomarkers for colorectal cancer. Clinical biochemistry.

[12] Wang JW, Peng SY, Li JT, Wang Y, Zhang ZP, Cheng Y, Cheng DQ, Weng WH, Wu XS, Fei XZ (2009). Identification of metastasis-associated proteins involved in gallbladder carcinoma metastasis by proteomic analysis and functional exploration of chloride intracellular channel 1. Cancer letters.

[13] Valenzuela SM, Mazzanti M, Tonini R, Qiu MR, Warton K, Musgrove EA, Campbell TJ, Breit SN (2000). The nuclear chloride ion channel NCC27 is involved in regulation of the cell cycle. The Journal of physiology.

[14] Tian Y, Guan Y, Jia Y, Meng Q, Yang J (2014). Chloride intracellular channel 1 regulates prostate cancer cell proliferation and migration through the MAPK/ERK pathway. Cancer biotherapy & radiopharmaceuticals.

[15] Li RK, Zhang J, Zhang YH, Li ML, Wang M, Tang JW (2012). Chloride intracellular channel 1 is an important factor in the lymphatic metastasis of hepatocarcinoma. Biomedicine & pharmacotherapy = Biomedecine & pharmacotherapie.

[16] Tung JJ, Kitajewski J (2010). Chloride intracellular channel 1 functions in endothelial cell growth and migration. Journal of angiogenesis research.

[17] Wang P, Zeng Y, Liu T, Zhang C, Yu PW, Hao YX, Luo HX, Liu G (2014). Chloride intracellular channel 1 regulates colon cancer cell migration and invasion through ROS/ERK pathway. World journal of gastroenterology : WJG.

[18] Wang P, Zhang C, Yu P, Tang B, Liu T, Cui H, Xu J (2012). Regulation of colon cancer cell migration and invasion by CLIC1-mediated RVD. Molecular and cellular biochemistry.

[19] Kang MK, Kang SK (2008). Pharmacologic blockade of chloride channel synergistically enhances apoptosis of chemotherapeutic drug-resistant cancer stem cells. Biochemical and biophysical research communications.

[20] Setti M, Savalli N, Osti D, Richichi C, Angelini M, Brescia P, Fornasari L, Carro MS, Mazzanti M, Pelicci G (2013). Functional role of CLIC1 ion channel in glioblastoma-derived stem/progenitor cells. Journal of the National Cancer Institute.

[21] Chang YH, Wu CC, Chang KP, Yu JS, Chang YC, Liao PC (2009). Cell secretome analysis using hollow fiber culture system leads to the discovery of CLIC1 protein as a novel plasma marker for nasopharyngeal carcinoma. Journal of proteome research.

[22] Tang HY, Beer LA, Chang-Wong T, Hammond R, Gimotty P, Coukos G, Speicher DW (2012). A xenograft mouse model coupled with in-depth plasma proteome analysis facilitates identification of novel serum biomarkers for human ovarian cancer. Journal of proteome research.

[23] Tang HY, Beer LA, Tanyi JL, Zhang R, Liu Q, Speicher DW (2013). Protein isoform-specific validation defines multiple chloride intracellular channel and tropomyosin isoforms as serological biomarkers of ovarian cancer. Journal of proteomics.

[24] Pisitkun T, Shen RF, Knepper MA (2004). Identification and proteomic profiling of exosomes in human urine. Proceedings of the National Academy of Sciences of the United States of America.

[25] Buschow SI, van Balkom BW, Aalberts M, Heck AJ, Wauben M, Stoorvogel W (2010). MHC class II-associated proteins in B-cell exosomes and potential functional implications for exosome biogenesis. Immunology and cell biology.

[26] Kesimer M, Scull M, Brighton B, DeMaria G, Burns K, O'Neal W, Pickles RJ, Sheehan JK (2009). Characterization of exosome-like vesicles released from human tracheobronchial ciliated epithelium: a possible role in innate defense. FASEB journal : official publication of the Federation of American Societies for Experimental Biology.

[27] Mathivanan S, Fahner CJ, Reid GE, Simpson RJ (2012). ExoCarta 2012: database of exosomal proteins, RNA and lipids. Nucleic acids research.

[28] Mathivanan S, Simpson RJ (2009). ExoCarta: A compendium of exosomal proteins and RNA. Proteomics.

[29] Staubach S, Razawi H, Hanisch FG (2009). Proteomics of MUC1-containing lipid rafts from plasma membranes and exosomes of human breast carcinoma cells MCF-7. Proteomics.

[30] Valadi H, Ekstrom K, Bossios A, Sjostrand M, Lee JJ, Lotvall JO (2007). Exosome-mediated transfer of mRNAs and microRNAs is a novel mechanism of genetic exchange between cells. Nature cell biology.

[31] Welton JL, Khanna S, Giles PJ, Brennan P, Brewis IA, Staffurth J, Mason MD, Clayton A (2010). Proteomics analysis of bladder cancer exosomes. Molecular & cellular proteomics : MCP.

[32] Sudol M, Chen HI, Bougeret C, Einbond A, Bork P (1995). Characterization of a novel protein-binding module—the WW domain. FEBS letters.

[33] Raiborg C, Rusten TE, Stenmark H (2003). Protein sorting into multivesicular endosomes. Current opinion in cell biology.

[34] Thery C, Amigorena S, Raposo G, Clayton A, Bonifacino Juan S. (2006). Isolation and characterization of exosomes from cell culture supernatants and biological fluids. Current protocols in cell biology.

[35] Kobayashi T, Vischer UM, Rosnoblet C, Lebrand C, Lindsay M, Parton RG, Kruithof EK, Gruenberg J (2000). The tetraspanin CD63/lamp3 cycles between endocytic and secretory compartments in human endothelial cells. Molecular biology of the cell.

[36] Soderberg O, Gullberg M, Jarvius M, Ridderstrale K, Leuchowius KJ, Jarvius J, Wester K, Hydbring P, Bahram F, Larsson LG (2006). Direct observation of individual endogenous protein complexes *in situ* by proximity ligation. Nature methods.

[37] Reya T, Morrison SJ, Clarke MF, Weissman IL (2001). Stem cells, cancer, and cancer stem cells. Nature.

[38] Ortensi B, Osti D, Pellegatta S, Pisati F, Brescia P, Fornasari L, Levi D, Gaetani P, Colombo P, Ferri A (2012). Rai is a new regulator of neural progenitor migration and glioblastoma invasion. Stem cells.

[39] Nickel W (2005). Unconventional secretory routes: direct protein export across the plasma membrane of mammalian cells. Traffic.

[40] Jiang L, Salao K, Li H, Rybicka JM, Yates RM, Luo XW, Shi XX, Kuffner T, Tsai VW, Husaini Y (2012). Intracellular chloride channel protein CLIC1 regulates macrophage function through modulation of phagosomal acidification. Journal of cell science.

[41] Valenzuela SM, Martin DK, Por SB, Robbins JM, Warton K, Bootcov MR, Schofield PR, Campbell TJ, Breit SN (1997). Molecular cloning and expression of a chloride ion channel of cell nuclei. The Journal of biological chemistry.

[42] Ulmasov B, Bruno J, Woost PG, Edwards JC (2007). Tissue and subcellular distribution of CLIC1. BMC cell biology.

[43] Tulk BM, Edwards JC (1998). NCC27, a homolog of intracellular Cl- channel p64, is expressed in brush border of renal proximal tubule. The American journal of physiology.

[44] Al-Nedawi K, Meehan B, Micallef J, Lhotak V, May L, Guha A, Rak J (2008). Intercellular transfer of the oncogenic receptor EGFRvIII by microvesicles derived from tumour cells. Nature cell biology.

[45] Jolliffe CN, Harvey KF, Haines BP, Parasivam G, Kumar S (2000). Identification of multiple proteins expressed in murine embryos as binding partners for the WW domains of the ubiquitin-protein ligase Nedd4. The Biochemical journal.

[46] Shirk AJ, Anderson SK, Hashemi SH, Chance PF, Bennett CL (2005). SIMPLE interacts with NEDD4 and TSG101: evidence for a role in lysosomal sorting and implications for Charcot-Marie-Tooth disease. Journal of neuroscience research.

[47] Behnke J, Eskelinen EL, Saftig P, Schroder B (2011). Two dileucine motifs mediate late endosomal/lysosomal targeting of transmembrane protein 192 (TMEM192) and a C-terminal cysteine residue is responsible for disulfide bond formation in TMEM192 homodimers. The Biochemical journal.

[48] Kelly BT, McCoy AJ, Spate K, Miller SE, Evans PR, Honing S, Owen DJ (2008). A structural explanation for the binding of endocytic dileucine motifs by the AP2 complex. Nature.

[49] Baietti MF, Zhang Z, Mortier E, Melchior A, Degeest G, Geeraerts A, Ivarsson Y, Depoortere F, Coomans C, Vermeiren E (2012). Syndecan-syntenin-ALIX regulates the biogenesis of exosomes. Nature cell biology.

[50] Henne WM, Stenmark H, Emr SD (2013). Molecular mechanisms of the membrane sculpting ESCRT pathway. Cold Spring Harbor perspectives in biology.

[51] Visvader JE (2011). Cells of origin in cancer. Nature.

[52] Visvader JE, Lindeman GJ (2012). Cancer stem cells: current status and evolving complexities. Cell stem cell.

[53] Milton RH, Abeti R, Averaimo S, DeBiasi S, Vitellaro L, Jiang L, Curmi PM, Breit SN, Duchen MR, Mazzanti M (2008). CLIC1 function is required for beta-amyloid-induced generation of reactive oxygen species by microglia. The Journal of neuroscience : the official journal of the Society for Neuroscience.

[54] Beznoussenko GV, Dolgikh VV, Seliverstova EV, Semenov PB, Tokarev YS, Trucco A, Micaroni M, Di Giandomenico D, Auinger P, Senderskiy IV (2007). Analogs of the Golgi complex in microsporidia: structure and avesicular mechanisms of function. Journal of cell science.

[55] Polishchuk RS, Polishchuk EV, Mironov AA (1999). Coalescence of Golgi fragments in microtubule-deprived living cells. European journal of cell biology.

