# Supplementary material for: Extracellular vesicle-mediated transfer of CLIC1 protein is a novel mechanism for the regulation of glioblastoma growth
Source: Oncotarget. 2015 Sep 30;6(31):31413–27. doi: 10.18632/oncotarget.5105 (PMC4741615; doi:10.18632/oncotarget.5105)
Supplement: Supplementary file 1 [file oncotarget-06-31413-s001.pdf]

SUPPLEMENTARY DATA

Cell culture

This study was approved by the Ethical Committee for human experimentation of IEO (European Institute of Oncology) and all patients signed an approved consent document prior to surgery. Surgical specimens of tumors were collected at the Neurosurgery Dpt. at IRCCS Istituto Clinico Humanitas and examined by a neuropathologist to verify that each case met criteria for GBM and to select a tissue fragment with high content of viable tumor tissue. Each tissue specimen was dissociated into single cell suspension in warmed EBSS (Earle's Balanced Salt Solution) containing papain (2 mg/ml) (Worthington Biochemical), EDTA (0.8 mg/ml) and L-Cystein (0.8 mg/ml) at 37°C for 1–2 hours. The dissociated tumor was filtered through a 70 μm filter and washed a minimum of three times prior culturing. GBM-derived CSCs were maintained as neurosphere in DMEM-F12 1:1 medium (Dulbecco's Modified Eagle Medium – Ham's F12 Nutrient Mixture) medium (Invitrogen) supplemented with B27 Supplement (Invitrogen), EGF (20 ng/ml), b-FGF (10 ng/ml) (PeproTech) and 2 μg/ml Heparin (Sigma), at 37°C in a 5% CO_2 humidified incubator. GBM CSC cultures were passaged by mechanically dissociation when spheres reached approximately 300–500 microns in diameter, and cell counts were performed at the time of passage. Human GBM cell lines U87MG, A172, LN405, U118MG, T98G, DBTRG-05MG and U373 MG were purchased from American Type Culture Collection (ATCC) and maintained in DMEM (Lonza) supplemented with 2 mM glutamine, 100 U/mL penicillin and 100 μg/mL streptomycin, and 10% FBS. All cell cultures were maintained at 37°C in a humidified 5% CO_2 incubator.

CLIC1 protein expression modulation

CLIC1 knock down was obtained by means of PLentiLox 3.7 lentiviral vector driving the expression of short hairpins specific for either human CLIC1 (siCLIC1) or firefly luciferase (NT), as described in (20). To overexpress CLIC1 protein, we excided CLIC1-FLAG cDNA from pIRES-eGFP plasmid (Clontech) (53) and cloned it into BamHI/BamHI sites of PLVX-Puro (Clontech) lentiviral vector (CLIC1 FLAG).

Lentivirus production and transduction

Recombinant lentiviruses were produced by transient transfection of 293T cells according to standard procedures. In brief, 293T cells were transfected with the vector, packaging plasmids, and a plasmid coding for the G protein of the vesicular stomatitis virus by the calcium-phosphate method. Virus was harvested 48 h later and concentrated by ultracentrifugation (68,000 × *g*). The multiplicity of infection (moi) was determined by infecting 293T cells, followed by flow cytometric quantification of eGFP-positive cells.

Lentiviral particles (multiplicity of infection = 1) targeting CLIC1 mRNA or bearing CLIC1-FLAG cDNA were added to the culture medium, along with 8 μg/mL polybrene. 48 hours after infection, GBM cells were incubated with 1 μg/mL puromycin for 3 days.

Electron microscopy

For routine electron microscopy (EM), purified EVs were fixed with 1% glutaraldehyde for 1 hour, washed, post-fixed with 1% reduced osmium tetroxide for 1 hour, washed, post-stained with 0,3% thiocarbohydrazide; refixed in the OsO4 and embedded into Epon. Ultrathin sections were placed on formvar-coated grids or slot-grids. Immune-EM analysis was performed as previously described (54, 55). Briefly, purified EVs were fixed with 1% glutaraldehyde and centrifuged. The pellet was embedded into gelatine and cryo-sections were prepared according to the standard procedure and cryo-sections were placed on slot-grid and labelled with antibodies against CLIC1 (rabbit polyclonal, 1:500, sc-134859 Santa Cruz, CA, USA) and CD63 (rabbit polyclonal, 1:1000, sc-15363, Santa Cruz, CA, USA) with subsequent labelling with protein A conjugated with 10 and 15 nm gold particles (UMC Utrecht, 1:60). Grids were observed at 200 kV with a Tecnai 20 electron microscope (FEI).

PKH26 labeling

Purified EVs were labeled with PKH26 (Sigma) according to manufacturer's instructions. Briefly, EVs (20 μg) were resuspended in 1 ml of diluent C, then mixed rapidly with a freshly prepared PKH26 solution in diluent C (final concentration during labeling step: 5×10^{-6} M) and incubated for 3 minutes at room temperature. The labeling step was stopped by addition of an equal volume of 1% BSA for 1 minute. After three washes in PBS by ultracentrifugation, EVs were resuspended in PBS (100 ng/μl).

EVs uptake assay

2×10^5 U87 MG cells were harvested in a 6-well plate with 500 μl of complete medium. Labeled EVs (1 μg) were diluted in the growth medium for the indicated times. The uptake was stopped by washing cells in cold PBS, followed by fixation in 4% paraformaldehyde (PFA). Cells were oserved under a confocal microscopy, and the

percentage of PKH-26 positive cells was assessed by FACS analysis.

Western blot analysis and immunoprecipitation

Primary antibodies: CLIC1 (mouse monoclonal, 1:1000, clone H-48, sc-134859, Santa Cruz, CA, USA), Vinculin (mouse monoclonal, 1:10000, clone HVIN-1, Sigma Aldrich, St. Louis, MO), CD63 (mouse monoclonal, 1:50, clone FC-5.01 18–7300 Invitrogen), tsg101 (goat polyclonal, 1:1000, sc-6037 Santa Cruz, CA,USA), GM130 (mouse monoclonal, 1:500, 610822, Becton Dickinson, Franklin Lakes, NJ, USA). GBM cells and EV samples were lysated on ice in 50–100 µl of lysis buffer (50 mM Tris–HCl buffer [pH 8], 10 mM $CaCl_2$, 5 mM EGTA [pH 8], 250 mM NaCl, Glycerol 10%, triton-x 100 1%) containing a cocktail of proteinase inhibitors (50 mM NAF, 10 mM NAPP, 10 mM NaOrtoV, PMSF [0.1 mg/ml], Leupeptin, Apoprotinin). Concentration of protein lysates was assessed by Bradford assay (Biorad, Hercules, CA). Each lysate (Whole cell extracts: 10 µg, EV lysates: 2 µg) was loaded onto a SDS-polyacrylamide gel electrophoresis (PAGE) under reducing conditions, and resolved proteins were transferred on to Nitrocellulose Transferring membranes (Protran ®, Indianapolis, IN) of 0.22-µm pore size. After blocking with 5% nonfat dry milk in Tris-Buffered Saline and Tween 20 (TBS-T [50 mM Tris, 150 mM NaCl, 0.05% Tween 20]), membranes were incubated overnight at 4°C with primary antibodies. Antibody binding was assessed by horseradish peroxidase (HRP)-conjugated secondary antibody (1:10000, Sigma Aldrich, St. Louis, MO). Immunoreactive bands were detected with ECL western blotting reagents (GE Healthcare Bio-Sciences, Pittsburgh,PA).

Immuno-precipitation was carried out with 20 µg of anti-GFP antibody (sc-9996, Santa Cruz, CA, USA) on 2 mg of whole cell extract overnight at 4°C. Membranes were blotted anti-CLIC1.

Apoptosis detection

For apoptosis analysis, cells were first fixed in 1% formaldehyde for 20 minutes on ice, washed once in PBS and fixed again in ethanol 75% for 30 minutes on ice. Fixed cells were incubated in Propidium Iodide (2.5 µg/ml) and RNAse (250 µg/ml) for 12 hours at +4°C and analyzed by flow cytometry.

MS-based proteomics analysis

EVs sample pellets from NT, CLIC1 FLAG and siCLIC1 cells were directly resuspended in Laemmli buffer and loaded on SDS–PAGE on a gradient gel (4–12% Tris–HCl Precast Gel, Invitrogen) for protein separation. Gels were stained with Colloidal Coomassie. Enzymatic in-gel digestion was performed essentially as previously described; briefly, samples were subjected to reduction in 10 mM DTT for 1 hat 56°C, followed by alkylation with 55 mM iodoacetamide for 45 min at RT, in the dark. Digestion was carried out saturating the gel with 12.5 ng/ml sequencing grade-modified trypsin (Promega) in 50 mM ammonium bicarbonate. After one overnight, peptide mixtures were acidified with tri-fluoro acetic acid (TFA, final concentration 3%), extracted from gel slices with two rounds of washes (in 30% acetonitrile (ACN)/3% TFA and then in 100% ACN, respectively). Extracted peptides were subsequently loaded onto home-made C18-Stage Tips, for concentration and desalting prior to LC-MS/MS analysis.

Liquid chromatography and tandem mass spectrometry (LC-MS/MS)

Peptide mixtures were separated by nano-liquid chromatography using an EASY-nLC system (ProxeonBiosystems, Odense, Denmark) connected to the hybrid dual-pressure linear ion trap/orbitrap mass spectrometer (LTQ OrbitrapVelos, Thermo Scientific). The nano-liter flow LC was operated in one column set-up with a 15 cm analytical column (75 µm inner diameter, 350 µm outer diameter) packed with C18 resin (ReproSil, Pur C18AQ 3 µm, Dr.Maisch, Germany). Solvent A was 0.1% FA and 5% ACN in ddH2O and solvent B was 95% ACN with 0.1% FA. Separation was performed with a gradient of 0–40% solvent B over 90 minutes, followed by a gradient of 40–60% for 10 minutes and 60–80% over 5 minutes at a flow rate of 250 nl/min. The LTQ OrbitrapVelos MS was used in the data-dependent mode. CID-fragmentation method when acquiring MS/MS spectra consisted of an orbitrap full MS scan followed by up to 10 LTQ MS/MS experiments (TOP10) on the most abundant ions detected in the full MS scan. Essential MS settings were as follows: full MS (AGC 1,000,000; resolution 30,000; m/z range 300–1500; maximum ion time 500 ms); MS/MS (AGC 30,000; maximum ion time 100 ms; minimum signal threshold 500; isolation width 2 Da; dynamic exclusion time setting 30 s (±10 ppm relative to the precursor ion m/z); singly charged ions and ions for which no charge state could be determined were excluded from selection. Normalized collision energy was set to 35%, and activation time to 10 ms; spray voltage, 2.2 kV; no sheath and auxiliary gas flow; heated capillary temperature, 275ºC; predictive automatic gain control (pAGC) enabled, and an S-lens RF level of 65%. For all full-scan measurements with the Orbitrap detector, a lock mass ion from ambient air (m/z 445.120024) was used as an internal calibrant.

Protein identification by MaxQuant software and data analysis

The mass spectrometric raw data were analyzed with the MaxQuant software (version 1.3.0.5) (http://www.maxquant.org/downloads.htm). A false discovery rate (FDR) of 0.01 for proteins and peptides, and a minimum peptide length of 6 aminoacids, were required. In order to improve mass accuracy of the precursor ions, the time-dependent recalibration algorithm of MaxQuant was used. The MS/MS spectra were searched by Andromeda engine against a human Uniprot sequence database (containing 86 725 protein sequences) combined with 262 common contaminants and concatenated with the reversed versions of all sequences. Enzyme specificity was set to Trypsin and maximum of two missed cleavages were allowed. Peptide identification was based on a search with an initial mass deviation of the precursor ion of up to 7 ppm. The fragment mass tolerance was set to 20 ppm on the m/z scale. Cysteine carbamidomethylation (Cys +57.021464 Da) was searched as fixed modification, whereas N-acetylation of protein (N-term, +42.010565 Da), oxidized Methionine (+15.994915 Da) were searched as variable modifications. Peptide and protein identification was performed automatically with MaxQuant using default settings for parameters. Briefly, peptide matches are assembled into protein groups. Posterior error probability (PEP) is calculated using Bayesian statistics as a probability of false hit using the peptide identification score and length of peptide. The protein group is assigned a PEP score by multiplying their peptide PEPs. Only peptides with distinct sequences and only highest scoring identified spectra were used. In the final list, the proteins identified were accepted only if they contain at least to two peptides, of which at least one unique (peptide > 1, unique > 0).

Gene ontology (GO) analysis

GO analysis was carried out by DAVID algorithm (http://david.abcc.ncifcrf.gov/) using the UNIPROT IDs obtained from the high confidence proteome identified for NT, CLIC1 FLAG and siCLIC1 EVs population. Particularly, the list of 2642 (NT), 2328 (CLIC1 FLAG) and 2310 (siCLIC1 EVs) were independently used as target list against a Human Uniprot sequence database (containing 86 725 protein sequences). GO terms were accepted as significantly enriched only when having a *P*-value < 0.01 (1%FDR) ,after correction for multiple testing using the Benjamini and Hochberg method (P*).

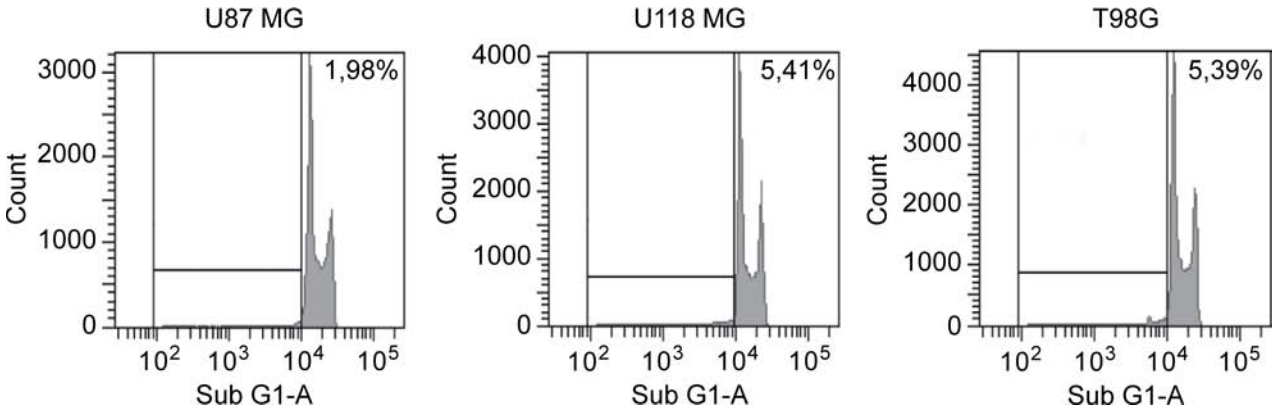

Supplementary Figure S1: The percentages of apoptotic cells were analysed in three different cell lines by PI staining for DNA content analysis by using flow cytometry (FACS).

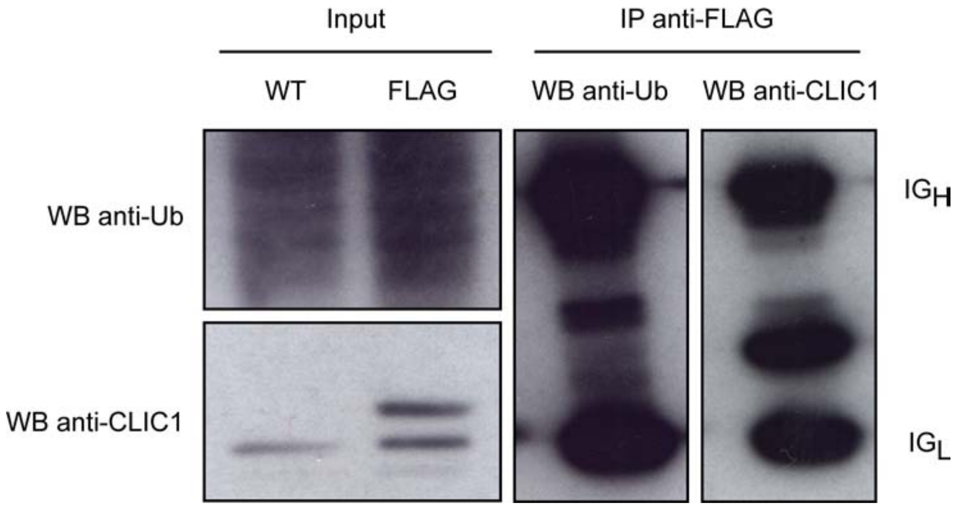

Supplementary Figure S2: CLIC1 protein ubiquitination. Whole cell lysates from U87 MG cells expressing the FLAG-tagged isoform of CLIC1 U87 MG were immunoblotted (Input), or immunoprecipitated against FLAG-tag (IP anti-FLAG) and then immunoblotted with ubiquitin antibody. The membrane was reprobed with CLIC1 antibody.

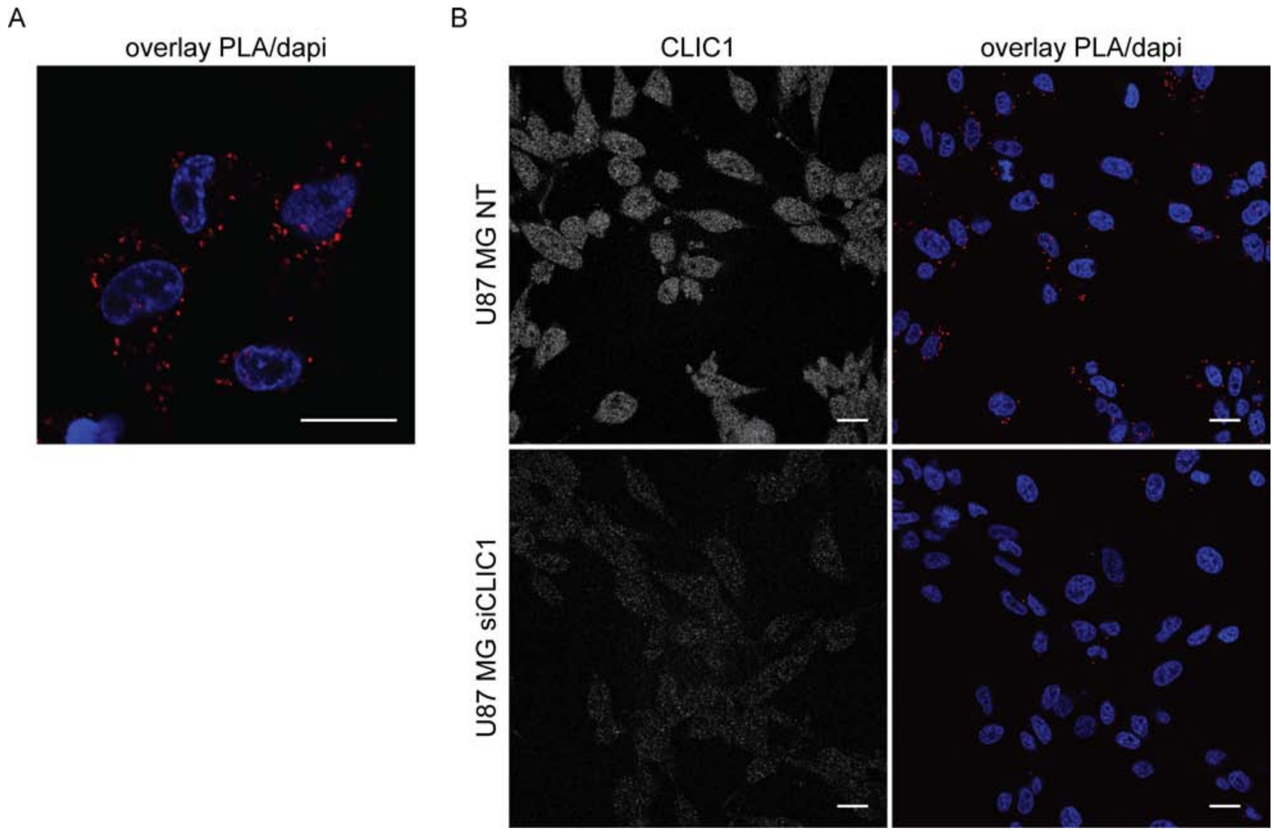

Supplementary Figure S3: A. CLIC1 and CD63 colocalization was assessed in U118 MG cells by in situ proximal ligation assay (PLA). **B.** For negative control, PLA assay was applied to CLIC1 and CD63 proteins in U87 MG control cells (U87 MG NT) and in CLIC1 silenced U87 MG cells (U87 MG siCLIC1). Scale bar, 20 μm. At least 10 cells from 5 sections were examined in three independent experiments.

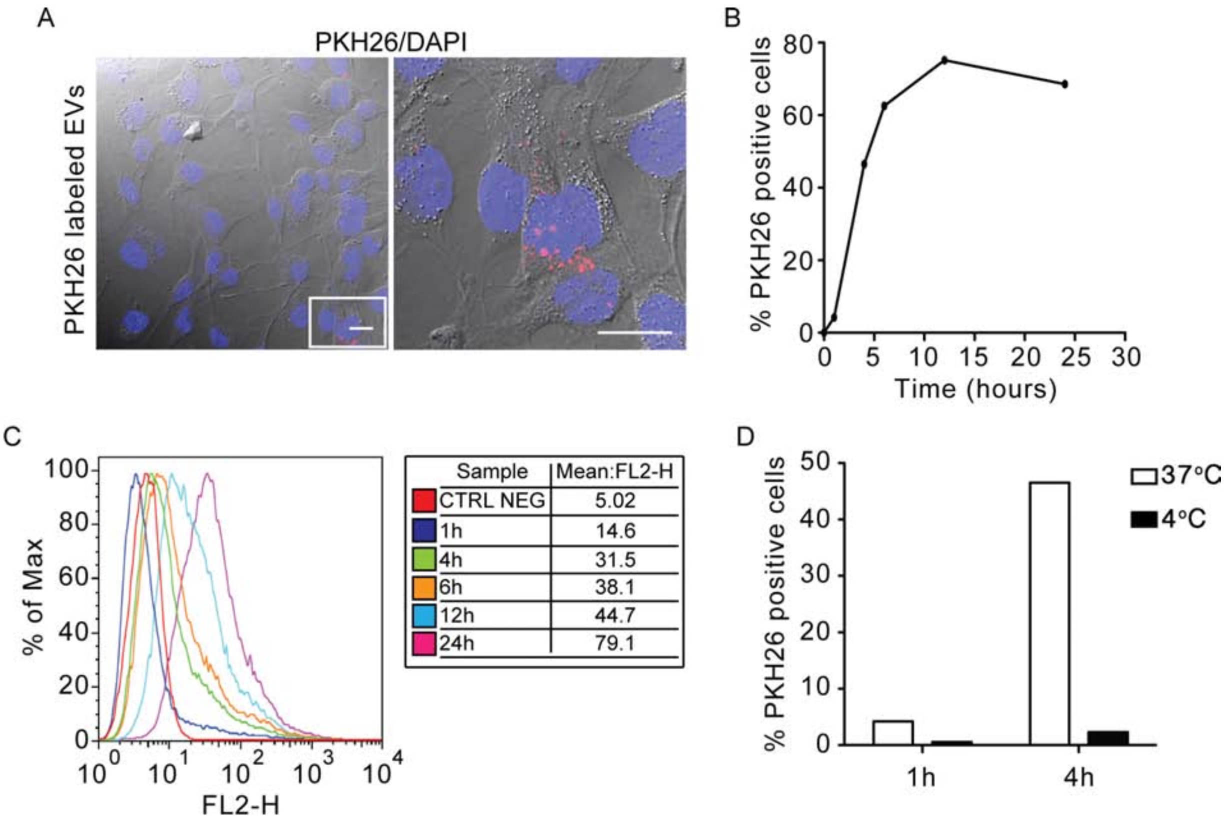

Supplementary Figure S4: GBM cell-derived EVs are efficiently internalized by recipient cells. A. Right, 293T cells were incubated with PKH26-labelled EVs and analyzed by confocal microscopy after 1 hour of incubation. Scale bar, 20 μm. The boxed area was enlarged. Red fluorescence spots in cell cytoplasm represent PKH26-labelled EVs. **B.** Quantification of EV internalization by 293T cells, expressed as percentage of PKH26 positive cells measured by FACS analysis at different time points, as indicated. **C.** Mean of fluorescence intensities relative to the time points assessed in B. **D.** Quantification of EV internalization by 293T cells, expressed as percentage of PKH26 positive cells, measured by FACS analysis 1 hour and 4 hours after EV incubation at 4°C and 37°C.

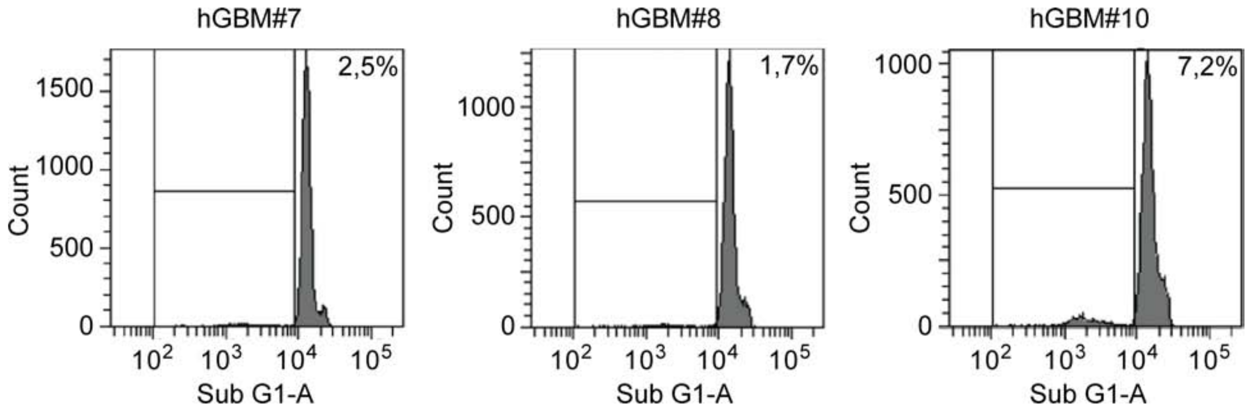

Supplementary Figure S5: The percentages of apoptotic cells in GBM CSCs were analysed by PI staining for DNA content analysis by using flow cytometry (FACS).

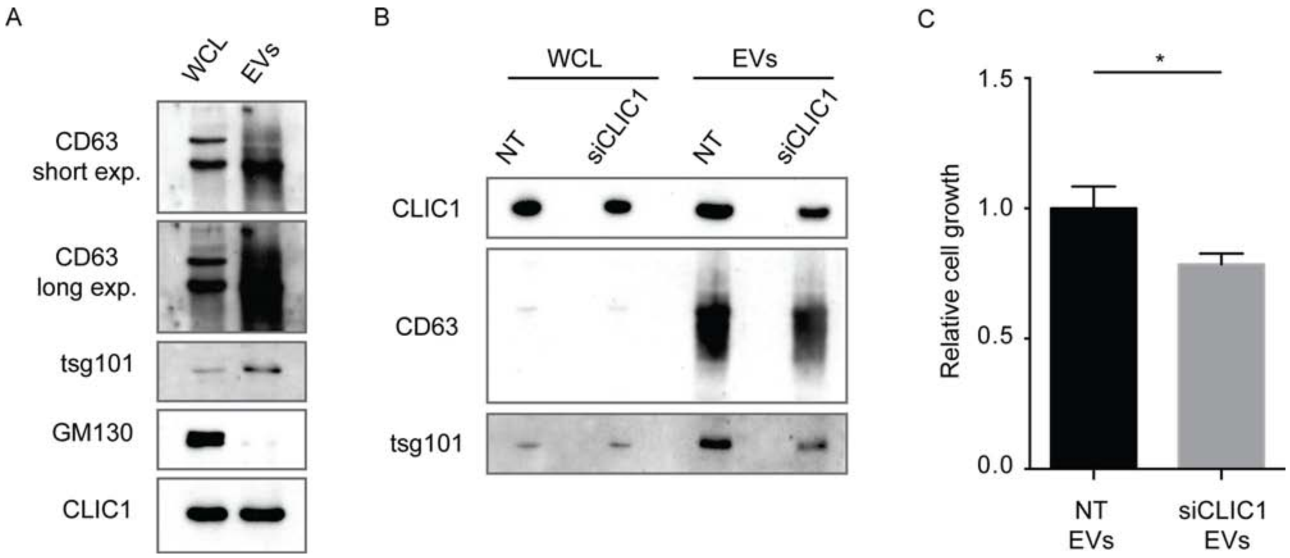

Supplementary Figure S6: CLIC1 secreted protein resides in EVs released from GBM CSCs. A. hGBM#23 CSCs whole cell lysates (WCL) and lysates from corresponding EVs were analysed for the indicated proteins by Western blotting. A representative immunoblot is shown. **B.** Whole cell lysates (WCL) obtained from control hGBM#23 CSCs (NT) and CLIC1 silenced hGBM#23 CSCs (siCLIC1), as well as lysates of corresponding EVs, were analysed by Western blotting using antibodies against CLIC1, and the EV markers CD63 and tsg101. A representative immunoblot is shown. **C.** hGBM#23 CSCs were cultured in presence of EVs (50 µg/ml) derived from either NT or siCLIC1 hGBM#23 CSCs for 120 hours and assessed for cell growth by 3-(4, 5-dimethylthiazol-2-yl)-2, 5-diphenyltetrazolium bromide (MTT) assay. Three independent experiments were performed; error bars represent standard error; $*p < 0.05$.

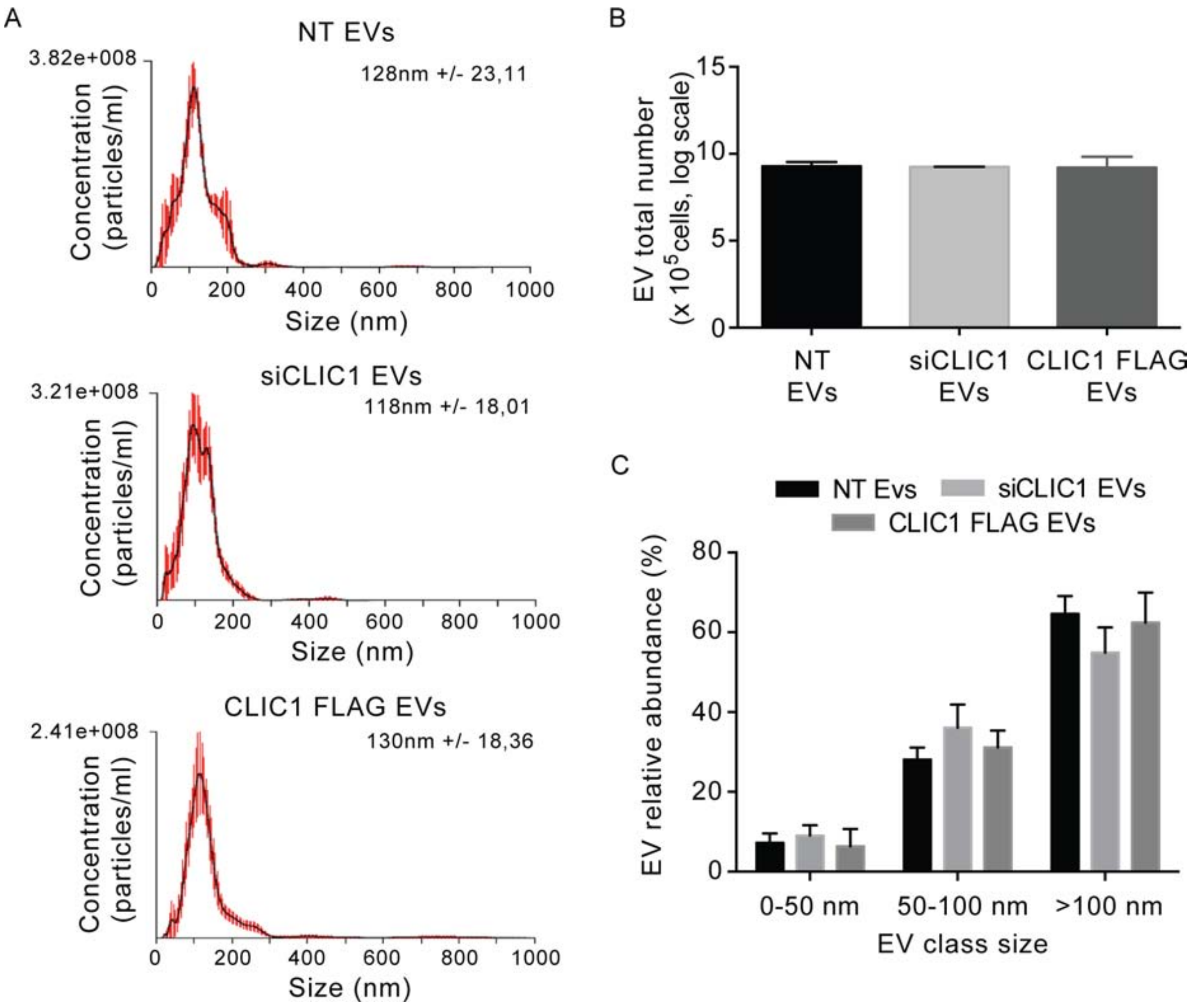

Supplementary Figure S7: EV size and yield are not affected by CLIC1 modulation in GBM cells. A. Size distribution of EVs shedded by T98G NT, T98G siCLIC1 and T98G CLIC1 FLAG cells measured by NTA. **B.** The yield of EVs shedded by T98G NT, T98G siCLIC1 and T98G CLIC1 FLAG cells measured by NTA. **C.** EVs were divided into three different dimensional classes, and their abundance was measured by NTA. Shown are the results from a representative experiment.

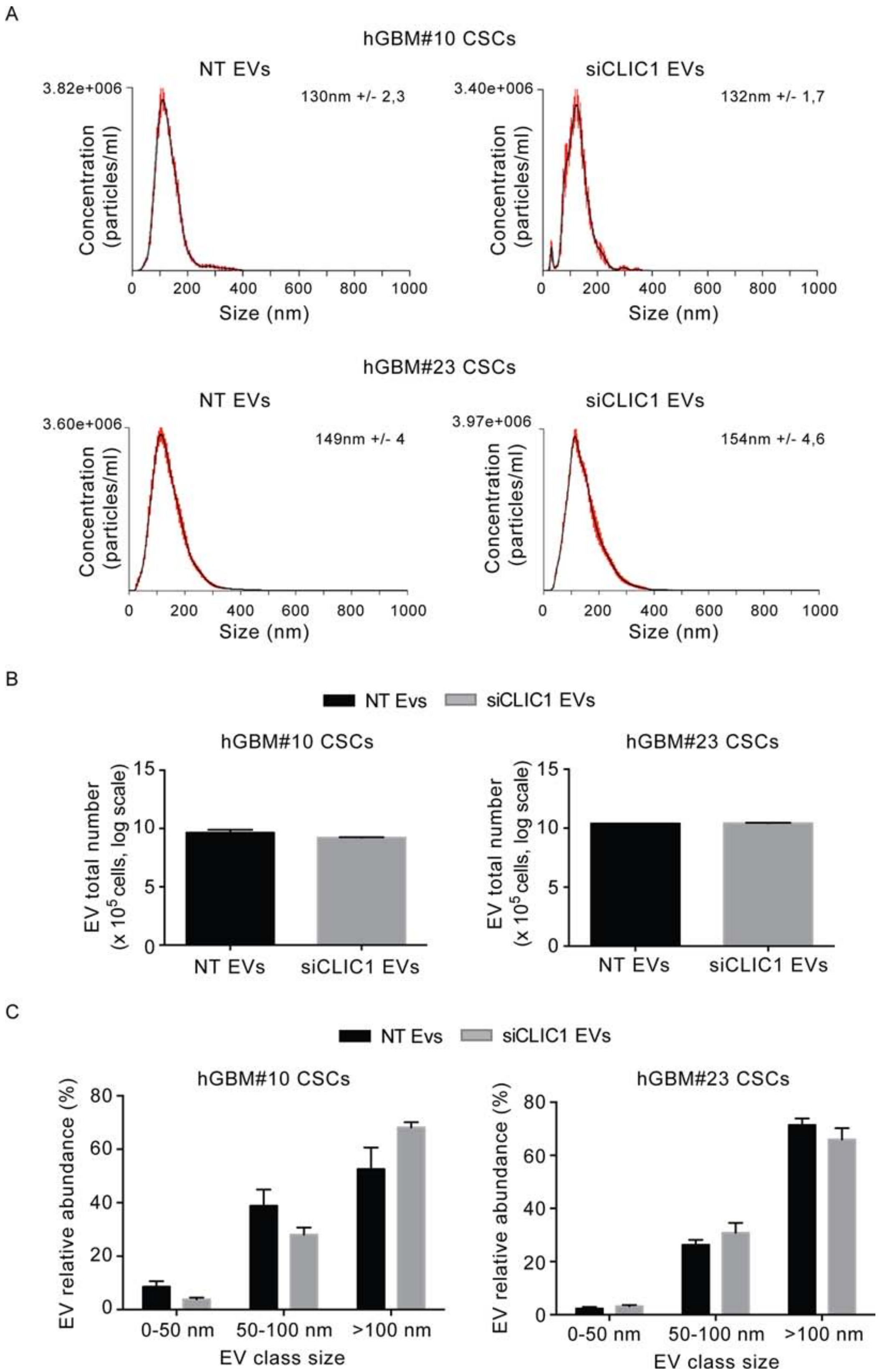

Supplementary Figure S8: EV size and yield are not affected by CLIC1 modulation in GBM CSCs. A. Size distribution of NT EVs and siCLIC1 EVs shedded by two different GBM patient-derived CSCs (hGBM#10 and hGBM#23), measured by NTA. **B.** The yield of EVs shedded by the two different GBM patient-derived CSCs as in A, measured by NTA. **C.** EVs were divided into three different dimensional classes, and their abundance was measured by NTA. Results from A - C illustrate a representative experiment.

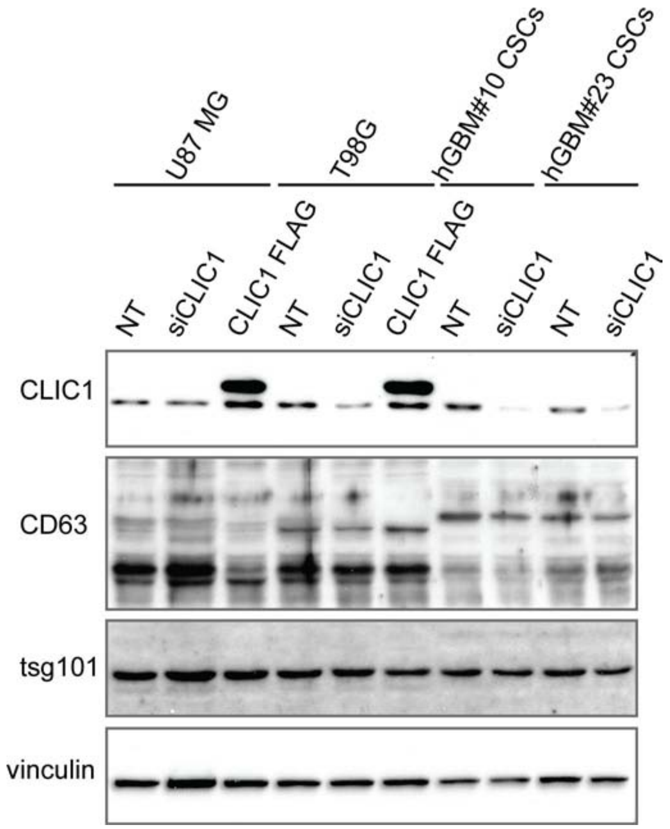

Supplementary Figure S9: CLIC1 modulation in GBM cells does not alter the expression of EV markers. The indicated GBM cells, either GBM cell lines (U87 MG and T98G) and GBM CSCs (hGBM#10 and hGBM#23), in which CLIC1 expression was silenced (siCLIC1) or overexpressed (CLIC1 FLAG), were analysed by Western blotting using antibodies against CLIC1 and EV markers CD63 and tsg101. Vinculin was used as loading control. A representative immunoblot is shown.

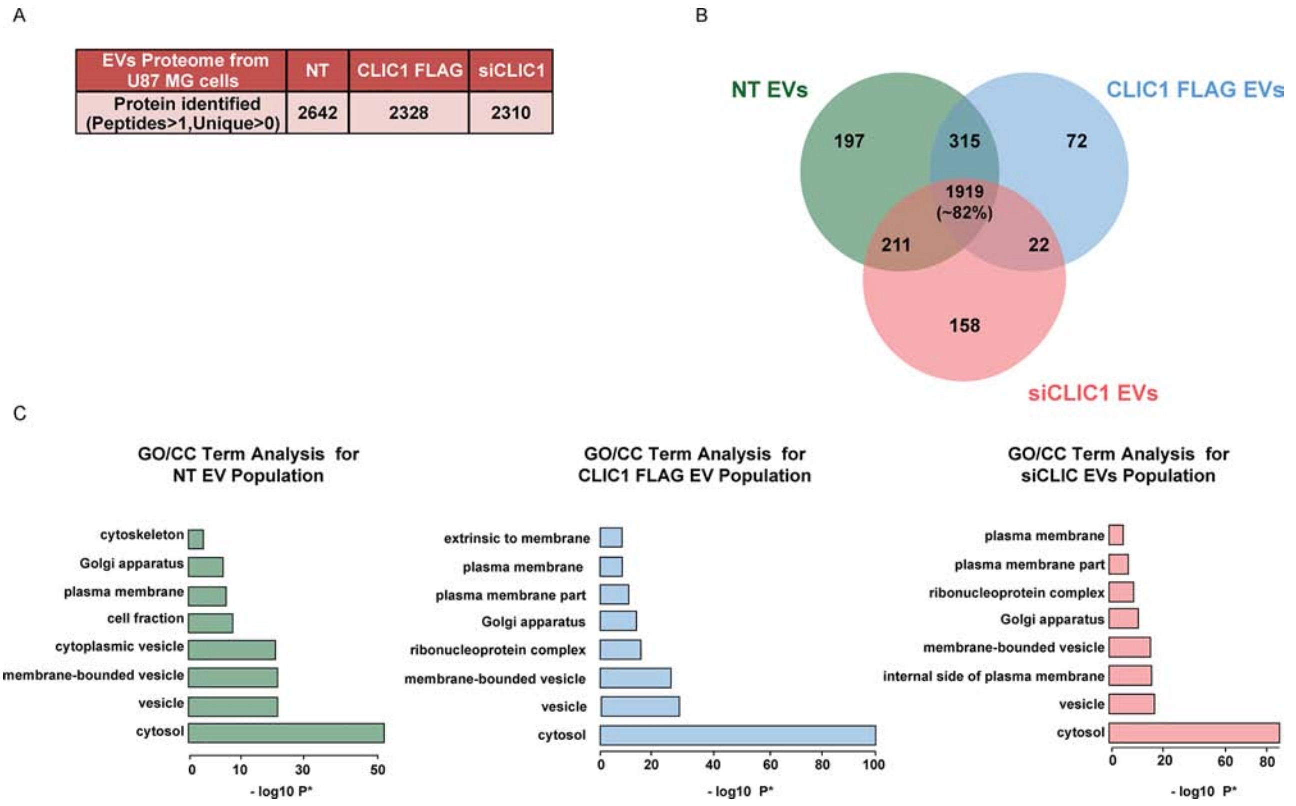

EVs Proteome from U87 MG cells	NT	CLIC1 FLAG	siCLIC1
Protein identified (Peptides>1,Unique>0)	2642	2328	2310

Supplementary Figure S10: Features of the EV proteomes from NT, siCLIC1and CLIC1 FLAG U87 MG cells. A. Table summarizing the numbers of proteins identified in each EV population (NT, CLIC1 FLAG and siCLIC1) upon LCMSMS analysis. Only the proteins identified with at least two peptides (peptides > 1), of which at least one unique (unique > 0), were selected. **B.** The Venn diagram indicated the high degree of overlap -with more than 80% of proteins in common- among the three EV samples. **C.** Gene ontology (GO) analysis for the proteins identified in the EVs isolated from NT (green) (lower left panel), CLIC1 FLAG (light blue) (lower center panel) and siCLIC1 (pink) (lower right panel) U87 MG cells. Cellular Components (CC) classification was performed using DAVID software (http://david.abcc.ncifcrf.gov/); only the terms with $P^* < 0.01$ (1% FDR) are reported, where P^* is the adjusted *P*-value applying the Benjamini and Hochberg correction.

Supplementary Table S1: MaxQuant software automatically generates the output table (proteingroup.txt) containing all proteins identified. A summary of the output table is reported in .excel format. Proteins identified in the NT, CLIC1 FLAG and siCLIC1 EVs population are listed in three individual spread-sheets (#1, #2, #3, respectively) upon filtering for high-confidence identification based on the assignment of at least 2 peptides (peptide > 1), at least one of which unique (unique > 0). In all the three cases, for each entry (raw), the corresponding Protein name, Gene name, Sequence coverage (%), Number of Peptides and Unique Peptides identified are reported, in the respective columns. MaxQuant automatically calculates the Posterior error probability (PEP) score during the identification process using pre-set FDR score (0.01).

Please see Supplementary Table S1
